# Supplementary material for: Simple Bayesian reconstruction and forecasting of stream water temperature for ecologists—A tool using air temperature, optionally flow, in a time series decomposition approach
Source: PLoS One. 2023 Sep 18;18(9):e0291239. doi: 10.1371/journal.pone.0291239 (PMC10506714; doi:10.1371/journal.pone.0291239)
Supplement: S1 File — (DOCX) [file pone.0291239.s001.docx]

# Supporting information

## Models’ priors

All the priors we used to fit our models are non informative relatively to their posteriors. To allow for the models to be easily applied onto other time series, the priors’ definition are based on mean and spread of the time series considered. $\mu_{90}$ and *IQ90* refer respectively to the average of a time series and to its 90% interquantile range. To avoid some confusion in parameter estimations, $\theta_{0^{'}}$ was defined as $\theta_{0}$ plus a positive deviation.

**S1Table. Priors used in the study**

| Model | Time series | Parameter | Prior |
| --- | --- | --- | --- |
| All | AT | $\alpha_{y}$ | $Normal\left( E=\mu_{AT},VAR=10 \right)$ |
| All | AT | $\beta_{y}$ | $LogNormal\left( E=log\left( IQ{90}_{AT}/2 \right)-.5,VAR=1 \right)$ |
| All | AT | $t_{0}$ | $Normal\left( E=N/4,CV=0.15 \right)$ |
| All | AT | $\rho$ | $Beta\left( \alpha=5,\beta=2 \right)$ |
| All | AT | $\tau$ | $Gamma\left( shape=2,rate=1 \right)$ |
| All | LFL | $\alpha_{y}$ | $Normal\left( E=\mu_{LFL},VAR=10 \right)$ |
| All | LFL | $\beta_{y}$ | $LogNormal\left( E=log\left( IQ{90}_{LFL}/2 \right)-.5,VAR=1 \right)$ |
| All | LFL | $t_{0}$ | $Normal\left( E=N/4-.5,CV=0.15 \right)$ |
| All | LFL | $\rho$ | $Beta\left( \alpha=5,\beta=2 \right)$ |
| All | LFL | $\sigma$ | $Gamma\left( shape=2,rate=1 \right)$ |
| All | WT | $\theta_{0}$ | $Normal\left( E=\mu_{WT}-IQ{90}_{WT}/2,VAR=10 \right)$ |
| All | WT | $\theta_{1}$ | $Normal\left( E=0,VAR=10 \right)$ |
| All | WT | $\theta_{2}$ | $Normal\left( E=0,VAR=10 \right)$ |
| All | WT | $\sigma_{min}$ | $Gamma\left( shape=2,rate=1 \right)$ |
| All | WT | $\theta'_{0}$ | $\theta_{0}+\Delta_{\theta_{0}}$ |
| All | WT | $\Delta_{theta_{0}}$ | $LogNormal\left( E=log\left( IQ{90}_{WT}/2 \right)-.5,VAR=1 \right)$ |
| All | WT | $\theta'_{1}$ | $Normal\left( E=0,VAR=10 \right)$ |
| All | WT | $\theta'_{2}$ | $Normal\left( E=0,VAR=10 \right)$ |
| All | WT | $\sigma_{max}$ | $Gamma\left( shape=2,rate=1 \right)$ |
| All | WT | $\sigma$ | $Gamma\left( shape=2,rate=1 \right)$ |
| $M_{1}$ | WT | $\rho$ | $Beta\left( \alpha=5,\beta=2 \right)$ |
| $M_{2}$ | WT | $\delta$ | $Normal\left( E=0,VAR=1 \right)$ |
| $M_{2}$ | WT | $\gamma$ | $Normal\left( E=0,VAR=1 \right)$ |
|  |  |  |  |

E, VAR, and CV refer respectively to the prior expected mean, variance and coefficient of variation. $\mu$ and $IQ90$ refer to the observed mean and the 90% interquantile range of the time series considered. N is the number of time steps within a year (i.e. 365 or 366).

## Time series used as application cases


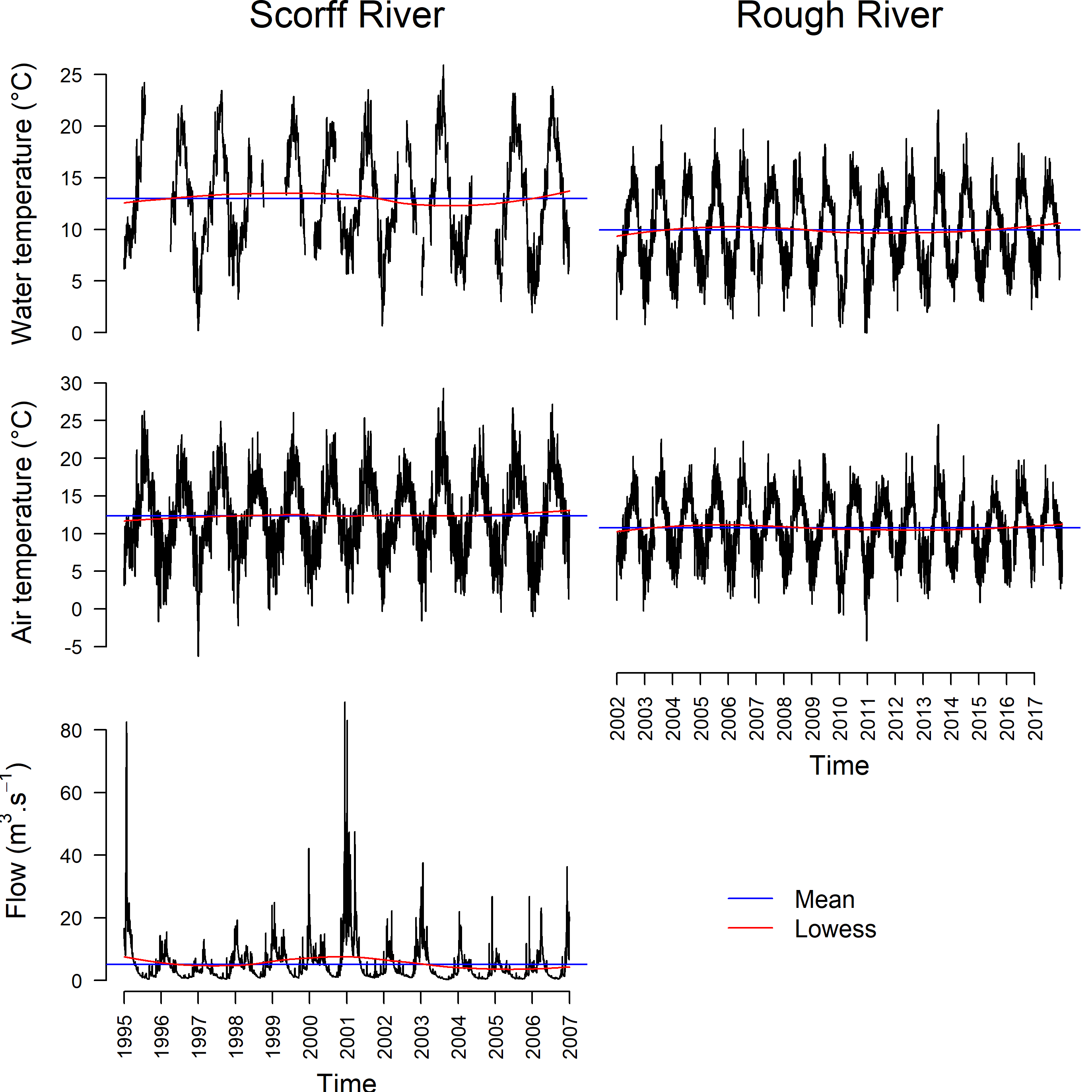


**S1 Fig. Times series or raw data from the Rough and Scorff rivers used in this study.**

## Fitted $\boldsymbol{\alpha}$ and $\boldsymbol{\beta}$ AT and Q time series


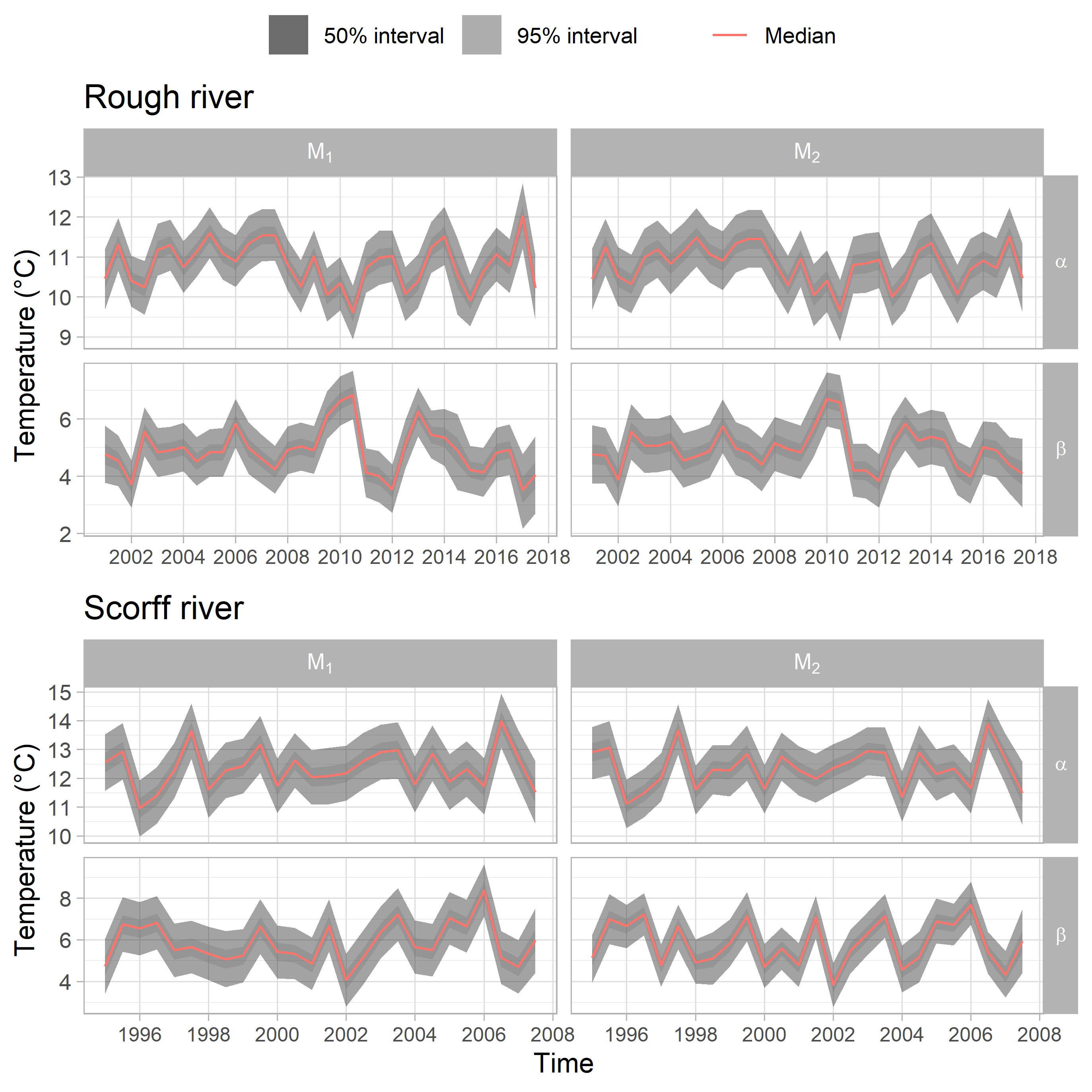


**S2 Fig. AT** $\boldsymbol{\alpha}$ **and** $\boldsymbol{\beta}$ **parameters posterior distributions from the fits of the models.**


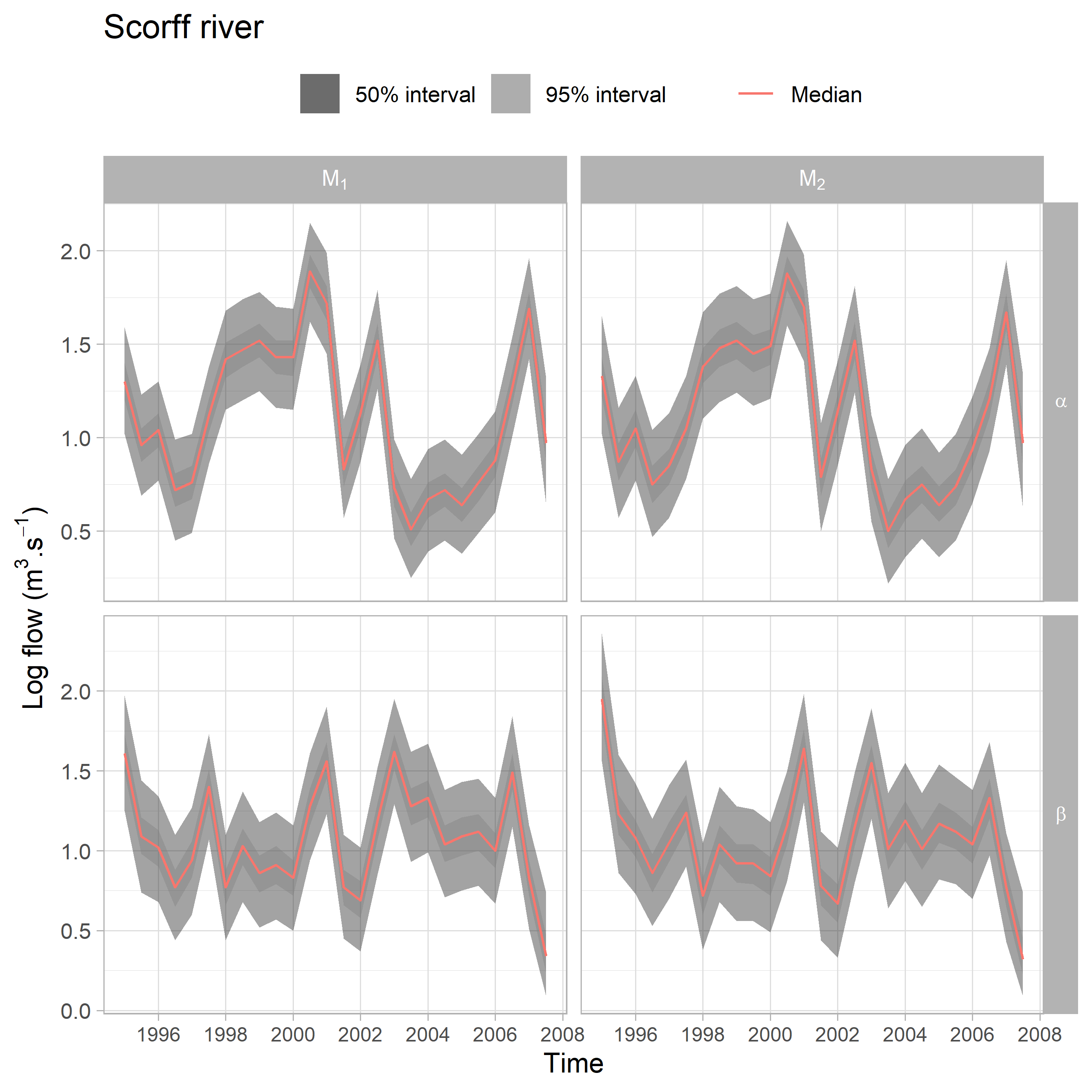


**S3 Fig. LFL** $\boldsymbol{\alpha}$ **and** $\boldsymbol{\beta}$ **parameters posterior distributions from the fits of the models.**
