# Supplementary material for: Simple Bayesian reconstruction and forecasting of stream water temperature for ecologists—A tool using air temperature, optionally flow, in a time series decomposition approach
Source: PLoS One. 2023 Sep 18;18(9):e0291239. doi: 10.1371/journal.pone.0291239 (PMC10506714; doi:10.1371/journal.pone.0291239)

PLOS ONE

Simple bayesian reconstruction and forecasting of stream water temperature for ecologists

--Manuscript Draft--

**Manuscript Number:**	PONE-D-23-03522
**Article Type:**	Research Article
**Full Title:**	Simple bayesian reconstruction and forecasting of stream water temperature for ecologists
**Short Title:**	Bayesian stream water temperature modeling
**Corresponding Author:**	Guillaume Bal, Ph.D.<br>Museum National d'Histoire Naturelle<br>Brunoy, FRANCE
**Keywords:**	water temperature; Freshwater; river; Bayesian modeling; Climate Change; uncertainty
**Abstract:**	Mitigating the impacts of global warming on wildlife entails four practical steps. First, we need to study how processes of interest vary with temperature. Second, we need to build good temperature scenarios. Third, processes can be forecast accordingly. Only then can we perform the fourth step, testing mitigating measures. While having good temperature data is essential, this is not straightforward for stream ecologists and managers. Water temperature (WT) data are often short and incomplete and future projections are currently not routinely available. There is a need for generic models which address this data gap with good resolution and current models are partly lacking. Here, we expand a previously published hierarchical Bayesian model that was driven by air temperature (AT) and flow (Q) as a second covariate. The new model can hindcast and forecast WT time series at a daily time step. It also allows a better appraisal of real uncertainties in the warming of water temperatures in rivers compared to the previous version, stemming from its hybrid structure between time series decomposition and regression. This model decomposes all-time series using seasonal sinusoidal periodic signals and time varying means and amplitudes. It then links the contrasted frequency signals of WT (daily and six month) through regressions to that of AT and optionally Q for better resolution. We apply this model to two contrasting case study rivers. For one case study, AT only is available as a covariate. This expanded model further improves the already good fitting and predictive capabilities of its earlier version while additionally highlighting warming uncertainties. The code is available online and can easily be run for other temperate rivers.
**Order of Authors:**	Guillaume Bal, Ph.D.
	Elvira De Eyto
**Additional Information:**	
**Question**	**Response**
**Financial Disclosure**<br><br>Enter a financial disclosure statement that describes the sources of funding for the work included in this submission. Review the submission guidelines for detailed requirements. View published research articles from *PLOS ONE* for specific examples.<br><br>This statement is required for submission and **will appear in the published article** if the submission is accepted. Please make	The author(s) received no specific funding for this work.

sure it is accurate.<br><br>**Unfunded studies**<br>Enter: *The author(s) received no specific funding for this work.*<br><br>**Funded studies**<br>Enter a statement with the following details:<br>• Initials of the authors who received each award<br>• Grant numbers awarded to each author<br>• The full name of each funder<br>• URL of each funder website<br>• Did the sponsors or funders play any role in the study design, data collection and analysis, decision to publish, or preparation of the manuscript?<br>• **NO** - Include this sentence at the end of your statement: *The funders had no role in study design, data collection and analysis, decision to publish, or preparation of the manuscript.*<br>• **YES** - Specify the role(s) played.<br><br>* typeset	
**Competing Interests**<br><br>Use the instructions below to enter a competing interest statement for this submission. On behalf of all authors, disclose any competing interests that could be perceived to bias this work—acknowledging all financial support and any other relevant financial or non-financial competing interests.<br><br>This statement is required for submission and **will appear in the published article** if the submission is accepted. Please make sure it is accurate and that any funding sources listed in your Funding Information later in the submission form are also declared in your Financial Disclosure statement.<br><br>View published research articles from *PLOS ONE* for specific examples.	The authors have declared that no competing interests exist.

**NO authors have competing interests**<br><br>Enter: *The authors have declared that no competing interests exist.*<br><br>**Authors with competing interests**<br><br>Enter competing interest details beginning with this statement:<br><br>*I have read the journal's policy and the authors of this manuscript have the following competing interests: [insert competing interests here]*<br><br>* typeset	
**Ethics Statement**<br><br>Enter an ethics statement for this submission. This statement is required if the study involved:<br><br>• Human participants<br>• Human specimens or tissue<br>• Vertebrate animals or cephalopods<br>• Vertebrate embryos or tissues<br>• Field research<br><br>Write "N/A" if the submission does not require an ethics statement.<br><br>General guidance is provided below. Consult the submission guidelines for detailed instructions. **Make sure that all information entered here is included in the Methods section of the manuscript.**	N/A

**Format for specific study types**<br><br>**Human Subject Research (involving human participants and/or tissue)**<br>• Give the name of the institutional review board or ethics committee that approved the study<br>• Include the approval number and/or a statement indicating approval of this research<br>• Indicate the form of consent obtained (written/oral) or the reason that consent was not obtained (e.g. the data were analyzed anonymously)<br><br>**Animal Research (involving vertebrate animals, embryos or tissues)**<br>• Provide the name of the Institutional Animal Care and Use Committee (IACUC) or other relevant ethics board that reviewed the study protocol, and indicate whether they approved this research or granted a formal waiver of ethical approval<br>• Include an approval number if one was obtained<br>• If the study involved *non-human primates*, add *additional details* about animal welfare and steps taken to ameliorate suffering<br>• If anesthesia, euthanasia, or any kind of animal sacrifice is part of the study, include briefly which substances and/or methods were applied<br><br>**Field Research**<br><br>Include the following details if this study involves the collection of plant, animal, or other materials from a natural setting:<br>• Field permit number<br>• Name of the institution or relevant body that granted permission	
**Data Availability**<br><br>Authors are required to make all data underlying the findings described fully available, without restriction, and from the time of publication. PLOS allows rare exceptions to address legal and ethical concerns. See the PLOS Data Policy and FAQ for detailed information.	No - some restrictions will apply

A Data Availability Statement describing where the data can be found is required at submission. Your answers to this question constitute the Data Availability Statement and **will be published in the article**, if accepted.<br><br>**Important:** Stating 'data available on request from the author' is not sufficient. If your data are only available upon request, select 'No' for the first question and explain your exceptional situation in the text box.<br><br>Do the authors confirm that all data underlying the findings described in their manuscript are fully available without restriction?	
**Describe where the data may be found in full sentences. If you are copying our sample text, replace any instances of XXX with the appropriate details.**<br><br>• If the data are **held or will be held in a public repository**, include URLs, accession numbers or DOIs. If this information will only be available after acceptance, indicate this by ticking the box below. For example: *All XXX files are available from the XXX database (accession number(s) XXX, XXX.*).<br>• If the data are all contained **within the manuscript and/or Supporting Information files**, enter the following: *All relevant data are within the manuscript and its Supporting Information files.*<br>• If neither of these applies but you are able to provide **details of access elsewhere**, with or without limitations, please do so. For example:<br><br>*Data cannot be shared publicly because of [XXX]. Data are available from the XXX Institutional Data Access / Ethics Committee (contact via XXX) for researchers who meet the criteria for access to confidential data.*<br><br>*The data underlying the results presented in the study are available from (include the name of the third party*	Most of the data used in this study will be provided in the repo 'https://github.com/GuillaumeBal/2023.bayes.stream.temp'. the only exception are the air temperature times series used for the Scorff river. Still, they can be requested for free on the french weather services website mentioned in the article.

Powered by Editorial Manager® and ProduXion Manager® from Aries Systems Corporation

and contact information or URL).<br>• This text is appropriate if the data are owned by a third party and authors do not have permission to share the data.<br><br>* typeset	
Additional data availability information:	Tick here if the URLs/accession numbers/DOIs will be available only after acceptance of the manuscript for publication so that we can ensure their inclusion before publication.

Powered by Editorial Manager® and ProduXion Manager® from Aries Systems Corporation

Manuscript

Click here to access/download;Manuscript;bal.&.deeyto.2022.bayes.stream.te

Simple bayesian reconstruction and forecasting of stream water temperature for ecologists

A tool using air temperature, optionally flow, in a time series decomposition approach

Guillaume Bal[1,*], Elvira de Eyto[2]

2022-12-01

Mitigating the impacts of global warming on wildlife entails four practical steps. First, we need to study how processes of interest vary with temperature. Second, we need to build good temperature scenarios. Third, processes can be forecast accordingly. Only then can we perform the fourth step, testing mitigating measures. While having good temperature data is essential, this is not straightforward for stream ecologists and managers. Water temperature (WT) data are often short and incomplete and future projections are currently not routinely available. There is a need for generic models which address this data gap with good resolution and current models are partly lacking. Here, we expand a previously published hierarchical Bayesian model that was driven by air temperature (AT) and flow (Q) as a second covariate. The new model can hindcast and forecast WT time series at a daily time step. It also allows a better appraisal of real uncertainties in the warming of water temperatures in rivers compared to the previous version, stemming from its hybrid structure between time series decomposition and regression. This model decomposes all-time series using seasonal sinusoidal periodic signals and time varying means and amplitudes. It then links the contrasted frequency signals of WT (daily and six month) through regressions to that of AT and optionally Q for better resolution. We apply this model to two contrasting case study rivers. For one case study, AT only is available as a covariate. This expanded model further improves the already good fitting and predictive capabilities of its earlier version while additionally highlighting warming uncertainties. The code is available online and can easily be run for other temperate rivers.

Keywords: water temperature, freshwater, river, Bayesian modeling, climate change, uncertainty

[1] PatriNat (OFB- MNHN), 91800 Brunoy, France (guillaume.bal.pro@gmail.com)

[2] Fisheries Ecosystems Advisory Services, Marine Institute, Furnace, Newport, Ireland (elvira.deeyto@marine.ie)

* Corresponding author

1 INTRODUCTION

Global warming is impacting ecological communities and ecosystems worldwide (Parmesan 2006; IPCC 2013; IPCC 2022). Temperature primarily impacts the physiology of organisms

(Bozinovic & Pörtner 2015), leading to changes in individual life history traits (Phillips *et al.* 2010; Bal *et al.* 2011; Dixie *et al.* 2015). Populations range(Parmesan & Yohe 2003; Thomas *et al.* 2006), phenology (Thackeray *et al.* 2010) and dynamics are also impacted (Post *et al.* 2009), steering disturbances in food webs and overall ecosystem change (Heino *et al.* 2009; Forrest 2016; Renner & Zohner 2018). Managing these ongoing and future changes is a major challenge for stakeholders.

Understanding and hence mitigating climate change impacts on biological communities involves four steps. First, we need to quantify the interactions between the process of interest and temperature. Second, we need to predict temperature under future greenhouse gas (GHG) scenarios. Third, we need to forecast our process under these future climate scenarios. Then, as the fourth step, we can work towards mitigating impacts, preferentially through and adaptive management framework (Williams 2011; Perry *et al.* 2020). Robust ecological and temperature data are essential cornerstones of this process.

Managing freshwater species conservation in the face of climate change is particularly challenging. First, such species are highly susceptible to climate change as they are mostly ectothermic and thus sensitive to temperature (Ormerod 2009; Jonsson & Jonsson 2009; Hamda *et al.* 2019). Shifts in spatial distribution are also constrained by the river network and habitat fragmentation (Ward *et al.* 2020).Second, water temperature time series are limited compared to air temperature time series. Indeed, water temperature time series are often quite short and prone to missing data, and unlike air temperature, are not generally included as outputs of climate change models. Generic models to complete and hindcast/forecast water temperature based on covariates such air temperature and flow are needed by ecologists and managers.

Different types of stream water temperature models exist but they present caveats to the ecologist (Bal *et al.* 2014). Process based models, e.g. energy budget models (Sinokrot & Stefan 1993; Webb & Zhang 2004; Garner *et al.* 2017) are realistic but complex. They require a lot of environmental and geological data, and are also location specific. This makes them of limited use for long term and large-scale forecasting. Statistical models on the other hand are less data demanding and rather simple (Benyahya *et al.* 2007). Examples of this

model type include regular time series models (AR, ARIMA), periodic autoregressive models, K nearest neighbors methods and neural network approaches (Chenard & Caissie 2008; Benyahya *et al.* 2011). These methods perform well for short term forecasting and filling short gaps. On the contrary, they perform poorly for long term predictions (Benyahya *et al.* 2007). The last category usually encountered is regressions models (Pilgrim *et al.* 1998; Pedersen & Sand-Jensen 2007; Zhu *et al.* 2018).While they may seem adequate, they can lead to significant biases in more long-term forecasting. Indeed, they do not disentangle long term trends, seasonality and short-term variations. An extensive review of stream water temperature models can be found in Caissie (2006) and allows for a better appraisal of the limitations exposed.

As a workaround, Bal *et al.* (2014) proposed a Bayesian hierarchical approach which was a hybrid between time series decomposition and regression. This approach only relied on water temperature together with air temperature and flow time series as covariates. It separated out long term, seasonal and short terms components of the time series and linked these components through correlation. This method has several advantages over regression, in that it outperformed correlation in both fit and forecasting capabilities, and appeared to be unbiased for long term forecasting. This hybrid approach also highlighted the true and high uncertainty in future river temperature warming. However, this approach lacked fine-scale modelling of deviations (ie daily) around long term and seasonal components, and requires additional refinement. Plainly, these short-term deviations were modeled using a first order autoregressive (AR1) process which limits fine hindcasting/forecasting abilities. Also, the model worked on 5-day average temperature. We here remedy this by i) working on daily temperature and ii) modeling daily deviations as depending on that of air temperature (AT) and flow (Q). Lastly, we offer the possibility of running this model without water flow data, as these are often not collected routinely. These new features, coupled with openly accessible code should be helpful to ecologists and stakeholders.

In this paper, we describe the fit and forecasting performances of this extended model and compare it to that of Bal *et al.* (2014) on two rivers with contrasting bio-geographical conditions and size. The Rough River is a small Irish spate river while the Scorff river is of

medium size and located in France. For the Rough river, air temperature is the only covariate. As in Bal *et al.* (2014), we use a fully hierarchical Bayesian framework. This provides a probabilistic rationale to quantify uncertainty in inferences and forecasting. which further ease out ecological analysis and risk management (Harwood & Stokes 2003).

2 MATERIAL AND METHOD

The new model version presented here is developed in reference to that of Bal *et al.* (2014). For this reason, we refer to the model in the previous publication as M_1 (for model 1). Our newest version described thereafter is called M_2.

2.1 Model M_2 structure

Model M_2 has a fully Bayesian hierarchical structures and produces estimates of water temperature (WT) based on air temperature (AT) and discharge (Q). This is achieved using three fully integrated modules. Module 1 desegregates WT, AR and Q time series into long term variations, seasonal fluctuations and short-term variations. Module 2 links WT components to that of the AT and Q (also referred to as covariates). Then, module 3 provides estimates of all unknown WT based on modules 1 and 2. Estimated WT can range anywhere from a few missing values up to several years in case of hindcasting or forecasting. The difference in model M_1 (Bal *et al.* 2014) and M_2 lies in Module 2 and so in the way daily variation in WT are modeled. In case of model M_1, daily variation was assumed to follow an autoregressive process. Here we expand module 2 to link these to those of the covariates.

Module 1 of the model M_2 desegregates WT and covariates time series (X_t in the following equation) as follow:

$$X_{y,t} = \alpha_y + \beta_y \times sin\left(\frac{2\pi}{n}(t - t_0)\right) + \epsilon_t \qquad (1)$$

α_y and β_y are the mean and amplitude of a time window. n is the number of time steps per year. In this article n is equal to the number of days within a year (365 or 366) while we set the time window y to 6 months as it offers a good tradeoff (see Bal *et al.* (2014)). t_0 sets the

seasonal signal on the year. For AT and Q, we modeled ϵ_t using first order autoregressive (AR1) processes centered on 0 (with autocorrelation coefficient ρ and standard deviation σ). Bal *et al.* (2014) modeled WT daily deviations the exact same way. This is where this new model differ and we detail this as part of the second module below.

Module 2 links WT time series to that AT and Q (if available). It comprises two sub-modules (2a and 2b). Sub-module 2a corresponds to the entirety of module 2 from Bal *et al.* (2014). In this first sub-module, we link parameter α_y and β_y of the WT time series to that of the covariates time series. To do so, we first reparametrized the sine signal within the time series using $max_y = \alpha_y + \beta_y$ and $min_y = \alpha_y - \beta_y$. Then we model WT maxima max_y^{WT} and minima min_y^{WT} as depending upon those of the covariates. Corresponding equations are:

$$\begin{gathered} max_y^{WT} \sim Normal\left(\mu_{max_y^{WT}}, \sigma_{max^{WT}}^2\right) \\ with\ \mu_{max_y^{WT}} = \theta_0 + \theta_1 \times max_y^{AT} + \theta_2 \times max_y^{Q} \\ min_y^{WT} \sim Normal\left(\mu_{min_y^{WT}}, \sigma_{min^{WT}}^2\right) \\ with\ \mu_{min_y^{WT}} = \theta_0 + \theta'_1 \times min_y^{AT} + \theta'_2 \times min_y^{Q} \end{gathered} \tag{2}$$

We kept this parametrization Bal *et al.* (2014) because of its conceptual sense. Let's assume that θ_1 is positive, θ_2 is negative and both θ'_1 and θ'_2 are positive. This corresponds to assuming that i) warm AT leads to warm WT, ii) high Q means warms WT in winter and cool it down in summer.

In addition, module 2b now links the short-term variations of WT to that of the covariates. We parametrized this addition to the original model M_1 as follows:

$$\begin{gathered} \epsilon_{y,t}^{WT} \sim Normal\left(\mu_{\epsilon_{y,t}^{WT}}, \sigma_{max^{WT}}^2\right) \\ \mu_{\epsilon_{y,t}^{WT}} = \delta \times \epsilon_{y,t}^{AT} + \gamma \times \epsilon_{y,t}^{Q} \times sin\left(\frac{2\pi}{n}(t - t_0 + n/2)\right) + \zeta_t^{WT} \end{gathered} \tag{3}$$

WT short term deviations thus are directly proportional to those of AT, although usually buffered. For Q, we included a sinusoidal component. This allows the impact of discharge deviations to change along the year. It shifts from positive in winter to negative in summer with a smooth transition along the year. We assume it as strictly antiphasing the WT

seasonal signal, hence the inclusion of $n\backslash 2$.Because we use the log discharge, small changes in summer flow have an impact more comparable to that of bigger changes in winter. We modeled the remaining errors (ζ_t^{WT}) using a white noise process.

Lastly, module 3 corresponds to the estimate of unknown temperatures. As our model is fully hierarchical, this module depends upon and integrates with modules 1 and 2. Consequently, the model propagates all uncertainties when estimating unknown WT based on covariates. In Bayesian software such BUGS, JAGS, STAN or NIMBLE (Hecht *et al.* (2021) and references therein), this is performed simultaneously to the fit. AT and Q times series just have to include hindcasting and/or forecasting parts if any. Module 1 also decomposes these 'extra' data while module 2 produces the WT estimates. This process is often referred to as deriving posterior predictive distributions.

2.2 Model application

To assess the performances of the model (M_2), we compare it model (M_1) on two study sites with contrasted sizes and environmental conditions and test both fit and forecasting capacities.

2.2.1 Assessing models' performances.

We checked the consistency of both models (M_1) and (M_2) a posteriori with the data using the χ^2 discrepancy statistics (Gelman *et al.* 2015). In particular, this posterior check allows to assess whether replicated data are similar to the original data. Its formula is:

$$\chi^2_{WT,\theta} = \sum_y \sum_t \frac{\left(WT_{t,y} - E\left(WT_{t,y}|\theta\right)\right)^2}{Var\left(WT_{t,y}|\theta\right)} \qquad (4)$$

where $E\left(WT_{y,t}|\theta\right)$ and $Var\left(WT_{y,t}|\theta\right)$ are the expected mean and variance of WT conditionally upon all parameters (θ). For each set of parameters drawn from the joint posterior distribution, we computed replicated and observed WT χ^2 values based on the equation. Then we computed a Bayesian p-value giving the probability that $\chi^2\left(WT_{rep}|\theta\right) >$ $\chi^2(WT_{obs}|\theta)$. Bayesian p-values close to 0.5 suggest congruency between the model and the

data. Very high or very low (about 0.95 or 0.05) values provide serious inconsistency warnings.

Secondly, we also compared the fitting performances of models M_1 and M_2 using the Deviance Information Criterion (DIC, Spiegelhalter *et al.* (2002)). DIC allows the comparison of goodness of fit while penalizing complexity in a way very similar to that of the Akaike Information Criterion. The smaller the DIC the better. A five points reduction is usually considered as indicative of a significant improvement.

Lastly, we checked the predictive performances of the models. For this we used cross a validation approach. Specifically, we used 2/3 of historical data as training set and forecast the last third. We used computed both the Root Mean Square Error (RMSE) and bias (proportions of under prediction) of the estimates.

2.2.2 Bayesian computations details

We implemented models M_1 and M_2 in JAGS (Plummer 2003) through R (Denwood 2016; R Core Team 2020). This software approximates the parameters posterior distributions using Monte Carlo Markov Chains (MCMC) algorithms. Specifically, we ran three MCMC in parallel for each model fit. We kept 10000 draws for each one after an initial burning period of 10000 draws. All diagnostics suggested the MCMC converged successfully. Priors used were all uninformative relative to the data. They are detailed in annex 7.1.

The code developed together with one of data-set are available on GitHub (https://github.com/GuillaumeBal/2023.bayes.stream.temperature). Readers can change the input data file with their own and run the model. Doing so do not require any in depth knowledge of both R and JAGS.

2.2.3 Study sites

Figure 1: Map of the application sites. Triangles correspond to water temperature and flow measurement stations while points indicate weather monitoring stations.

WT, AT and Q time series come from the Rough (Mayo, Ireland) and Scorff (Brittany, France) rivers (1. Both rivers support long-term environmental monitoring and fisheries related research (Bagliniere *et al.* 2005; Montorio *et al.* 2018; de Eyto *et al.* 2020). They

contrast in size, location as well as available data (Table 1). In particular, reliable Q time series are lacking on the Rough river. This allowed for testing the performances of our approach with only one covariate.

The Scorff River flows into the Atlantic Ocean (1. INRA staff members measure daily WT at the Moulin des Princes station with Tidbit data loggers, precision of 0.2°C). Daily AT come from the Lorient (Lan Bihoue) airport meteorological station, about 9 km away from the water measurement station, and were provided by the French weather services (https://donneespubliques.meteofrance.fr/). Q records come from 8 km upstream of the Moulin des Princes station and were downloaded from a national public portal (https://hydro.eaufrance.fr/). The tree time series are 13 years long (1995 to 2007) and 9.6% data are missing. Diadromous fish population from this river are extensively monitored and studied (https://www6.rennes.inrae.fr/u3e_eng/ABOUT-US/Organisation/The-ORE-DiaPFC-monitoring-unit)

The Rough River (also called the Srahrevagh River) is a tributary of the Srahmore River in the Burrishoole Catchment. The Srahmore flows into Lough Feeagh which is connected to the saline coastal lagoon Lough Furnace. The entire system discharges into Clew Bay on the Atlantic Ocean (1, data cover the period 2002-2016). Daily WT was measured using a StowAway TidbiT temperature data logger from Onset (TB132-05+37). Air temperature data was recorded at the Newport (Furnace) manual weather station (jointly run by Met Éireann and the Marine Institute, station 833). Maximum and minimum temperatures are recorded using mercury thermometers, and average daily temperature is taken as the average of these two readings. Data were downloaded from www.met.ie, licensed under a Creative Commons Attribution 4.0 International (CC BY 4.0) License.

Some more details about the catchments are provided in table 1. Raw times series are available in annex 7.2.

Table 1: Application sites and data sets description.

River	Rough	Scorff
Location	Mayo, Ireland	Brittany, France

River	Rough	Scorff
Mouth (Latitude & Longitude)	53°97'N, -9°57'W	47°28'N, 3°23' W
Drainage area (km^2)	4.50	480
River Length (km)	12.3	75
Estuary length (km)	NA	15
Source altitude (m above sea level)	370	270
Geology (predominant)	Quartzite & schist	Granite & schist
% agricultural	NA	60
% woodland	NA	30
% peat bog	3.2	NA
% forestry	32.20	NA
% natural grassland	7.9	NA
Climate	Temperate oceanic	Mild oceanic
Precipitations(mm)	~1560	~1000
Water temperature period	2002 – 2016	1995 - 2007
Water temperature mean (°C)	9.91	12.88
Water temperature missing data (%)	0.82	26.98
Air temperature period	1960 – 2016	1995 – 2007
Air temperature mean (°C)	10.25	12.39
Air temperature missing data (%)	0.21	0.63
Water discharge period	NA	1995 – 2007
Water discharge mean ($m^3.s^{-1}$)	NA	4.95
Water discharge missing data (%)	NA	1.05

2.2.4 Forecasting under climate change

We used model M_1 and M_2 full joint posteriors to predict WT temperature based on AT warming projections. In particular, we picked a range of AT warming of 0 to 5°C according

to the latest IPCC estimates (IPCC 2021), excluding the most extreme warming scenario. We did this for several reasons. First, Bal *et al.* (2014) revealed WT warming should be lower than that of AT and quite uncertain. We wanted to check whether this result was robust to the updated time step and model structure. We also wanted to see whether two rather contrasted rivers would differ in their response.

3 RESULTS

The results section is composed of four parts. First, we show differences in fitted series of α and β with both models. Then, we focus on quantitative results showing how the updated approach of modelling the water temperature residuals as depending upon those of air temperature and flow outperformed the version with a simple AR1 process, both for forecasting and fitting. Third, we look at parameters of model M_2 and highlight how those shared with the first model evolved to better understand model differences. Lastly, we showcase forecasting and missing value estimates from model M_2.

3.1 Seasonal variation in the time series

Comparing time series of fitted α and β between models M_1 and M_2 provides basic insights into differences in their fit and behavior. The ranges of values are greater for model M_2 than for model M_1 whatever the parameter series and river considered (Figure 2). This can be highlighted by statistically significant changes in the variance of median values. For instance, the variance of the median estimates of the α_{WT} posterior series on the Scorff river was 0.48 for model M_2 versus only 0.17 with model M_2 (p.value = 0.012, Fischer test). While series ranges differed, mean values of median estimates for all series were equivalent. The same was observed for average 95 and 50% credible intervals.

In case of AT and Q, α and β time series derived using both models were quite similar (Figures 9 and 10 in annex 7.3).Mean values, variances and 95 and 50% credible intervals of the time series deviated from each other only fractionally. Only a few posteriors, such as the last couple of the Rough river α_{AT}, were obviously different.

Figure 2: WT α and β parameters posteriors for model fit. In case of model M_2 WT residuals are linked to those of AT and Q.

3.2 Fit and predictive performances comparison

Overall, results suggest better predictive performances for model M_2 on both rivers. In addition, fit statistics that tie to the good predictive performances described in our also favored of model M_2.

The only exception to the general superior performances of model M_2 are DIC values (Table 2). Indeed, differences of several hundred of points in deviance in favor of model M_1 were highlighted by the analysis. The same was observed for DIC values. The higher model complexity of model M_1, highlighted by pD values, did not change this outcome. This general difference resulted from the highest fit flexibility linked to modeling the residuals with an AR1 process in case of model M_1. We also noted that the lack of covariate data tends to increase pD values in case of model M_2 as observed for the Rough river.

Table 2: Fitting and forecasting performances summary statistics

River	Model	RMSE	Chi2	Deviance	PD	DIC
Rough	M_2	1.81	0.50	16664.69	381.62	17046.31
Rough	M_1	2.35	0.44	15589.20	31.20	15620.40
Scorff	M_2	1.86	0.50	10763.39	46.75	10810.15
Scorff	M_1	2.87	0.40	7334.66	29.16	7363.82

Discrepancies between 6-month averages of replicated data and observed water temperature were clearly in favor of the more complex model M_2 (Figure 3). Indeed, related posterior distribution for M_2 appeared both better centered on zero but also exhibited a much narrower credible interval. For the Scorff river, the average 95% interval dropped from 1.63 to 0.48. For the Rough River, the corresponding values decreased from 1.00 to 0.40. We can also that the distributions for the second half of years 2009 and 2010 on the Rough River are far from being centered on 0 in case of the old model M_1 (Figure 3). We can observe the same problem with the second half of years 1996 and 2006 on the Scorff river.

Figure 3: Boxplot of discrepancies between observed and fitted mean six month temperature. Model M_1 features an AR1 modeling of WT residuals. In case of model M_2, WT residuals are linked to those of AT and optionally Q.

RMSE values clearly highlighted the better forecasting performances of model M_2. Indeed, incorporating the link between residuals when modeling water temperature allowed for a reduction in RMSE of about 30 and 40% for the Rough and Scorff River respectively (table 2). The forecasting capabilities of model M_2 are further highlighted on figure 4. Here, we selected a small portion of historical water temperature data from each river, containing both low and high daily variation. On both rivers, observed and predicted daily variations follow each other closely. High frequency changes are well captured by model M_2, as can be seen for early summer (days 75-100) for both rivers (Figure 4). The average 50% credible interval around predictions is about 0.85°C for both rivers, while the 95% credible interval spans slightly more than 5°C (Figure 4).

Figure 4: Observed versus forecasted temperatures coming from the cross validation. Yellow lines correspond to observed WT temperatures. The blue line is the median value of predictive posteriors. Grey areas indicate 95% and 50% intervals of the forecasts.

3.3 Posteriors of parameters linking water temperature to the covariates

Modeling the link between the residuals of the different time series resulted in significant differences in θ posterior distributions (Figure 5). Indeed, three main type of changes were noticeable. First, posteriors of θ parameters tightened. This was particularly significant on the Scorff River with, for instance, reduction of about 50% in the 95% credible of parameters $\theta_{1'}$, θ_2 and $\theta_{2'}$. Second, posteriors of θ_1 and $\theta_{1'}$ shifted toward stronger positive values. With model M_2, the credible intervals of parameters linked to LFL were well centered on 0 compared to that of model M_1. Meanwhile, the uncertainty around the regression appears greater with model M_2 than with model M_1 (see σ posteriors, Figure 5)

Figure 5: Comparison of the posteriors of θ parameters from equation set (2) updated with both model M_1 and M_1. The left hand side of the figure are for parameters linking max_y^{WT} to

max_y^{AT} and optionaly min_y^{Q}. The left hand side of the figure are for parameters linking min_y^{WT} to min_y^{AT} and optionaly max_y^{Q}.

Parameters δ and γ linking daily variations in water temperature to that of the covariates were estimated as positive together with tight posterior distributions on both rivers (Figure 6). All posteriors were centered on values comprise between 0.5 and 0.6 suggesting changes in water temperature to be buffered when compared to those of covariates. The posterior of parameter γ appeared about 10 times wider than that of δ on the Scorff river (0.279 vs 0.029 95% credible), suggesting less precision into the impact of flow.

Figure 6: Posteriors of model M_2 parameters linked to daily variations in water temperature. Details can be found in equation set (3).

3.4 Forecast warming

Model M_2 forecast WT greater warming than model M_1 on both rivers (Figure 7). These differences are directly attributable to the differences in θ posteriors detailed above (Figure 5). Consequently, the difference between models was greater on the Scorff river. The WT median warming forecast by model M_2 on the Scorff closely followed that of AT to peak at 4.61°C for an AT warming of 5°C (median value), indicating that AT warming is still likely to be a bit faster than WT warming there. Meanwhile, model M_1 median warming forecast was only 1.33°C and its 95% credible interval barely overlapped that of model M_2. For the Rough river, the WT warming forecasts greatly overlapped between models although model M_2 suggested it could be slightly faster. Both suggested that warning WT warming should be close to AT warming. Lastly, WT warming forecast coming from model M_2 were more uncertain than with model M_1. The average 95% credible of forecast WT warming were of 1.88 and 2.01°C on the Scorff river with model M_1 and model M_2 respectively for a 5°C AT warming. On the Rough river, corresponding values were of 1.24 and 1.61°C.

Figure 7: Warming of stream water temperature forecasted across a range of air temperature warming. The dotted blue line is the 1:1 line.

4 DISCUSSION

Expanding the time series decomposition-based approach of Bal *et al.* (2014) to better model daily variation in WT in addition to seasonal variations lead to greatly improved WT forecasting abilities. In doing so, RMSE decreased by about 30% on the Rough river and about 40% on the Scorff River. Discrepancies between 6-month averages of replicated data and observed water temperature were also better with the new M_2 model than with the previous M_1 model. This is indicative of a better fit as replicated data generated under model M_2 are more similar to observed data. It also means forecast or hindcast 6-month means should be more reliable. The use of an AR1 structure to model WT residual as in model M_1 is very flexible and allows for capturing variability in fit. However, this can be considered as on over-fit when considering our goals. Indeed, model M_1 was less successful than M_2 at producing long term hindcasts or projected forecasts (Figure 4).

Interestingly, the new model suggested that daily variation in flow may be more important to WT than seasonal variation. Indeed, in case of our new model, the θ parameters linking the seasonal component of WT to that of flow on the Scorff river appeared centered on 0, meaning that they may not be statistically significant. This appeared surprising as AT and Q six months minima and maxima time series were almost identical between models (See annex 7.3). In addition, the correlations between Q and AT times series entering the set of equations (2) remained rather weak, suggesting that the signals were contrasted between covariates. Correlation were even slightly decreasing when going from model M_1 to M_2, changing from 0.47 to 0.4 for min_{AT} and max_Q, and from -0.38 to -0.30 for max_{AT} and min_Q. It would be interesting to see if this behavior is also observed on other rivers. Still, the impact of daily flow variations on WT has practical consequences. It indicates that daily management of flow to avoid extremely low and high flow might be useful in the context of climate change adaptation (Arismendi *et al.* 2013). In particular, ensuring adequate flow in summer will serve to alleviate thermal stress, as flow can mitigate warming. The main issue is that both rainfall prediction by climate model and the subsequent rainfall-runoff model may lack enough precision (Koch & Grunewald 2010).

Model M_2 also highlighted that WT warming should track that of AT closer than predicted with model M_1. In case of the Scorff river, WT potential warming appeared to be at the same scale as that of the AT warming scenario. In contrast, the Rough River WT forecast warming increased to a lesser degree and projections were below those for AT. The improvements in forecast observed on the Scorff River is likely linked to two key points. First it appears that modeling the residuals as done with model M_2 allows for a more precise estimate of the long-term component of WT times series than when using model M_1. The AR1 structure used in M_1 probably absorbed a part of the signal in 6-month values. The Scorff river WT times series was also relatively short and prone to a lot of missing values. Those two things together probably led to an even greater parameter confusion while fitting the signal decomposition. Thus, we observed even more uncertainty in the 6 months min and max estimates from model M_1 on the Scorff compared to those derived from model M_2. Lastly, a rather important WT warming on the Scorff river is also congruent with the energy budget theory (Caissie 2006). The Scorff river is much longer than the Rough river (75 and 12.3 km respectively). As such, thermal exchanges between air and water are more important and some homogenization is to be expected. Headwaters may thus be way less impacted by climate change as observed on the Rough river and in the USA (Luce *et al.* 2014). Consequently, their role in mitigating the global warming impact could be important. We note, however, that the opposite observation about the response of headwaters to warming has also been made (Hrachowitz *et al.* 2010) and further analyses is necessary.

The overall uncertainty associated with WT warming is high. This will have to be accounted for in decision making. Indeed, whatever the model, the 95% credible interval in WT warming was about 3°C around a given temperature. IPCC scenarios highlight uncertainties in AT warming in 2100 close to 3.5°C (IPCC 2021). The combined uncertainty is very high even over the medium term. Integrating it in a probabilistic framework is important for future ecological studies and decisions making (Harwood & Stokes 2003). The approach we developed makes this possible.

Further extensions of our model are possible. For now, the model is limited to one station per river, but may be extended to several. This has already been done for instance with models based on linear (Hrachowitz *et al.* 2010) and non linear regressions (Johnson *et al.*

2014). The application could also encompass several rivers as done for instance with regression or neural network (DeWeber & Wagner 2014). Such an approach could include a hierarchical structure to test for an increased link between AT and WT as the stations are further downstream. The number and length of the time series would have to be chosen carefully as Bayesian fit can be slow when dealing with a large amount of data. Other covariates may also be included in further research. Shading by riparian vegetation can significantly alter WT and vegetation may change due to climate or land use management decisions (Macedo *et al.* 2013; Garner *et al.* 2017). The shading factor could be included within the modelling of residuals as reducing extreme values through and additional exponential multiplier.

We offer stream ecologists and stakeholders a generic, parsimonious and effective way to complete, hindcast and forecast WT time series in a temperate context. Using only AT and optionally Q time series, it predicts daily WT. The Bayesian framework allows for a full propagation of uncertainties while doing so. A probabilistic rational can then be used for management decision. The code is available on GITHUB (https://github.com/GuillaumeBal/2023.bayes.stream.temperature) so readers can perform their own fits. Providing correctly formatted data (locally observed WT, AT and optionally flow data) and the specification a few model options is all that is required to run model both M_1 and M_2.

5 ACKNOWLEDGEMENTS

We are grateful to the staff of the Marine Institute, of the 'Unite Expérimentale d'Ecologie et d'Ecotoxicologie Aquatique', of Hydroportail and of Météo France for collecting and providing the data used in this study.

6 REFERENCES

Arismendi, I., Safeeq, M., Johnson, S.L., Dunham, J.B. & Haggerty, R. (2013). Increasing synchrony of high temperature and low flow in western North American streams: Double trouble for coldwater biota? *Hydrobiologia*, **712**, 61–70.

Bagliniere, J.L., Marchand, F. & Vauclin, V. (2005). Interannual changes in recruitment of the Atlantic salmon (Salmo salar) population in the River Oir (Lower Normandy, France):

Relationships with spawners and in-stream habitat. *Ices Journal of Marine Science*, **62**, 695–707.

Bal, G., Rivot, E., Baglinière, J.-L., White, J. & Prévost, E. (2014). A Hierarchical Bayesian Model to Quantify Uncertainty of Stream Water Temperature Forecasts. *PLOS ONE*, e115659.

Bal, G., Rivot, E., Prevost, E., Piou, C. & Bagliniere, J.L. (2011). Effect of water temperature and density of juvenile salmonids on growth of young-of-the-year Atlantic salmon Salmo salar. *Journal of Fish Biology*, **78**, 1002–1022.

Benyahya, L., Caissie, D., St-Hilaire, A., Ouarda, T.B.M.J. & Bobée, B. (2007). A Review of Statistical Water Temperature Models. *Canadian Water Resources Journal / Revue canadienne des ressources hydriques*, **32**, 179–192.

Benyahya, L., St-Hilaire, A., Quarda, T.B.M.J., Bobée, B. & Ahmadi-Nedushan, B. (2011). Modeling of water temperatures based on stochastic approaches: Case study of the Deschutes River. *Revue du génie et de la science de l'environnement*.

Bozinovic, F. & Pörtner, H.-O. (2015). Physiological ecology meets climate change. *Ecology and Evolution*, **5**, 1025–1030.

Caissie, D. (2006). The thermal regime of rivers: A review. *Freshwater Biology*, **51**, 1389–1406.

Chenard, J.F. & Caissie, D. (2008). Stream temperature modelling using artificial neural networks: Application on Catamaran Brook, New Brunswick, Canada. *Hydrological Processes*, **22**, 3361–3372.

Denwood, M.J. (2016). runjags: An R package providing interface utilities, model templates, parallel computing methods and additional distributions for MCMC models in JAGS. Journal of Statistical Software, 71, 1–25.

DeWeber, J.T. & Wagner, T. (2014). A regional neural network ensemble for predicting mean daily river water temperature. *Journal of Hydrology*, **517**, 187–200.

Dixie, B., White, H. & Hassall, M. (2015). Effects of microclimate on behavioural and life history traits of terrestrial isopods: Implications for responses to climate change. *ZooKeys*, 145–157.

de Eyto, E., Connolly, P., Dalton, C., Jennings, E. & McGinnity, P. (2020). The Burrishoole catchment: A western salmonid stream-lake system. *Ireland's Rivers*, pp. 357–381. UCD Press, Dublin.

Forrest, J.R. (2016). Complex responses of insect phenology to climate change. *Current Opinion in Insect Science*, **17**, 49–54.

Garner, G., Malcolm, I.A., Sadler, J.P. & Hannah, D.M. (2017). The role of riparian vegetation density, channel orientation and water velocity in determining river temperature dynamics. *Journal of Hydrology*, **553**, 471–485.

Gelman, A., Carlin, J.B., Stern, H.S., Dunson, D.B., Vehtari, A. & Rubin, D.B. (2015). Bayesian Data Analysis, 3rd edn. Chapman; Hall/CRC, New York.

Hamda, N.T., Martin, B., Poletto, J.B., Cocherell, D.E., Fangue, N.A., Van Eenennaam, J., Mora, E.A. & Danner, E. (2019). Applying a simplified energy-budget model to explore the effects of temperature and food availability on the life history of green sturgeon (Acipenser medirostris). *Ecological Modelling*, **395**, 1–10.

Harwood, J. & Stokes, K. (2003). Coping with uncertainty in ecological advice: Lessons from fisheries. *Trends in Ecology & Evolution*, **18**, 617–622.

Hecht, M., Weirich, S. & Zitzmann, S. (2021). Comparing the MCMC Efficiency of JAGS and Stan for the Multi-Level Intercept-Only Model in the Covariance- and Mean-Based and Classic Parametrization. *Psych*, **3**, 751–779.

Heino, J., Virkkala, R. & Toivonen, H. (2009). Climate change and freshwater biodiversity: Detected patterns, future trends and adaptations in northern regions. *Biological Reviews*, **84**, 39–54.

Hrachowitz, M., Soulsby, C., Imholt, C., Malcolm, I.A. & Tetzlaff, D. (2010). Thermal regimes in a large upland salmon river: A simple model to identify the influence of landscape controls and climate change on maximum temperatures. *Hydrological Processes*, **24**, 3374–3391.

IPCC. (2013). *Climate change 2013: The physical science basis. Contribution of working group I to the fifth assessment report of the intergovernmental panel on climate change*. Cambridge University Press, Cambridge, United Kingdom; New York, NY, USA.

IPCC. (2021). *Climate Change 2021: The Physical Science Basis. Contribution of Working Group I to the Sixth Assessment Report of the Intergovernmental Panel on Climate Change [Masson-Delmotte, V., P. Zhai, A. Pirani, S.L. Connors, C. Péan, S. Berger, N. Caud, Y. Chen, L. Goldfarb, M.I. Gomis, M. Huang, K. Leitzell, E. Lonnoy, J.B.R. Matthews, T.K. Maycock, T. Waterfield, O. Yelekçi, R. Yu, and B. Zhou (eds.)].*

IPCC. (2022). Climate Change 2022: Impacts, Adaptation, and Vulnerability. Contribution of Working Group II to the Sixth Assessment Report of the Intergovernmental Panel on Climate Change [H.-O. Pörtner, D.C. Roberts, M. Tignor, E.S. Poloczanska, K. Mintenbeck, A. Alegría, M. Craig, S. Langsdorf, S. Löschke, V. Möller, A. Okem, B. Rama (eds.)].

Johnson, M.F., Wilby, R.L. & Toone, J.A. (2014). Inferring air–water temperature relationships from river and catchment properties. *Hydrological Processes*, **28**, 2912–2928.

Jonsson, B. & Jonsson, N. (2009). A review of the likely effects of climate change on anadromous Atlantic salmon Salmo salar and brown trout Salmo trutta, with particular reference to water temperature and flow. *Journal of Fish Biology*, **75**, 2381–2447.

Koch, H. & Grunewald, U. (2010). Regression models for daily stream temperature simulation: Case studies for the river Elbe, Germany. *Hydrological Processes*, **24**, 3826–3836.

Luce, C., Staab, B., Kramer, M., Wenger, S., Isaak, D. & McConnell, C. (2014). Sensitivity of summer stream temperatures to climate variability in the Pacific Northwest. *Water Resources Research*, **50**, 3428–3443.

Macedo, M.N., Coe, M.T., DeFries, R., Uriarte, M., Brando, P.M., Neill, C. & Walker, W.S. (2013). Land-use-driven stream warming in southeastern Amazonia. *Philosophical Transactions of the Royal Society B: Biological Sciences*, **368**, 20120153.

Montorio, L., Evanno, G. & Nevoux, M. (2018). Intra- and interspecific densities shape life-history traits in a salmonid population. *Oecologia*, **188**, 451–464.

Ormerod, S.J. (2009). Climate change, river conservation and the adaptation challenge. *Aquatic Conservation-Marine and Freshwater Ecosystems*, **19**, 609–613.

Parmesan, C. (2006). Ecological and evolutionary responses to recent climate change. *Annual Review of Ecology Evolution and Systematics*, **37**, 637–669.

Parmesan, C. & Yohe, G. (2003). A globally coherent fingerprint of climate change impacts across natural systems. *Nature*, **421**, 37–42.

Pedersen, N.L. & Sand-Jensen, K. (2007). Temperature in lowland Danish streams: Contemporary patterns, empirical models and future scenarios. *Hydrological Processes*, **21**, 348–358.

Perry, D.C., Chaffee, C., Wigand, C. & Thornber, C. (2020). Implementing adaptive management into a climate change adaptation strategy for a drowning New England salt marsh. *Journal of Environmental Management*, **270**, 110928.

Phillips, B.L., Brown, G.P. & Shine, R. (2010). Life-history evolution in range-shifting populations. *Ecology*, **91**, 1617–1627.

Pilgrim, J.M., Fang, X. & Stefan, H.G. (1998). Stream temperature correlations with air temperatures in Minnesota: Implications for climate warming. *Journal of the American Water Resources Association*, **34**, 1109–1121.

Plummer, M. (2003). JAGS: A program for analysis of Bayesian graphical models using Gibbs sampling. pp. 20–22.

Post, E., Brodie, J., Hebblewhite, M., Anders, A.D., Maier, J.A.K. & Wilmers, C.C. (2009). Global Population Dynamics and Hot Spots of Response to Climate Change. *BioScience*, **59**, 489–497.

R Core Team. (2020). *R: A language and environment for statistical computing*. Vienna, Austria.

Renner, S.S. & Zohner, C.M. (2018). Climate Change and Phenological Mismatch in Trophic Interactions Among Plants, Insects, and Vertebrates. *Annual Review of Ecology, Evolution, and Systematics*, **49**, 165–182.

Sinokrot, B.A. & Stefan, H.G. (1993). STREAM TEMPERATURE DYNAMICS - MEASUREMENTS AND MODELING. *Water Resources Research*, **29**, 2299–2312.

Spiegelhalter, D.J., Best, N.G., Carlin, B.R. & Linde, A. van der. (2002). Bayesian measures of model complexity and fit. *Journal of the Royal Statistical Society Series B-Statistical Methodology*, **64**, 583–616.

Thackeray, S.J., Sparks, T.H., Frederiksen, M., Burthe, S., Bacon, P.J., Bell, J.R., Botham, M.S., Brereton, T.M., Bright, P.W., Carvalho, L., Clutton-Brock, T., Dawson, A., Edwards, M., Elliott, J.M., Harrington, R., Johns, D., Jones, I.D., Jones, J.T., Leech, D.I., Roy, D.B., Scott, W.A., Smith, M., Smithers, R.J., Winfield, I.J. & Wanless, S. (2010). Trophic level asynchrony in rates of phenological change for marine, freshwater and terrestrial environments. *Global Change Biology*, **16**, 3304–3313.

Thomas, C.D., Franco, A.M.A. & Hill, J.K. (2006). Range retractions and extinction in the face of climate warming. *Trends in Ecology & Evolution*, **21**, 415–416.

Ward, A.S., Wondzell, S.M., Schmadel, N.M. & Herzog, S.P. (2020). Climate Change Causes River Network Contraction and Disconnection in the H.J. Andrews Experimental Forest, Oregon, USA. *Frontiers in Water*, **2**.

Webb, B.W. & Zhang, Y. (2004). Intra-annual variability in the non-advective heat energy budget of Devon streams and rivers. *Hydrological Processes*, **18**, 2117–2146.

Williams, B.K. (2011). Adaptive management of natural resources—framework and issues. *Journal of Environmental Management*, **92**, 1346–1353.

Zhu, S., Nyarko, E.K. & Hadzima-Nyarko, M. (2018). Modelling daily water temperature from air temperature for the Missouri River. *PeerJ*, **6**, e4894.

7 Annex

7.1 Models' priors

All the priors we used to fit our models are non informative relatively to their posteriors. To allow for the models to be easily applied onto other time series, the priors' definition are based on mean and spread of the time series considered. μ_{90} and $IQ90$ refer respectively to the average of a time series and to its 90% interquantile range. To avoid some confusion in parameter estimations, $\theta_0{}'$ was defined as θ_0 plus a positive deviation.

Table 3: Priors used in the study. E, VAR, SD, tau, and CV refer respectively to the prior expected mean, variance and coefficient of variation μ and $IQ90$ refer to the observed mean and the 90% interquantile range of the time series considered. N is the number of time steps within a year (i.e. 365 or 366).

Model	Time.series	Parameter	Prior
All	AT	α_y	$Normal(E = \mu_{AT}, VAR = 10)$
All	AT	β_y	$LogNormal(E = log(IQ90_{AT}/2) - .5, VAR = 1)$
All	AT	t_0	$Normal(E = N/4, CV = 0.15)$
All	AT	ρ	$Beta(\alpha = 5, \beta = 2)$
All	AT	τ	$Gamma(shape = 2, rate = 1)$
All	LFL	α_y	$Normal(E = \mu_{LFL}, VAR = 10)$
All	LFL	β_y	$LogNormal(E = log(IQ90_{LFL}/2) - .5, VAR = 1)$
All	LFL	t_0	$Normal(E = N/4 - .5, CV = 0.15)$
All	LFL	ρ	$Beta(\alpha = 5, \beta = 2)$
All	LFL	σ	$Gamma(shape = 2, rate = 1)$
All	WT	θ_0	$Normal(E = \mu_{WT} - IQ90_{WT}/2, VAR = 10)$
All	WT	θ_1	$Normal(E = 0, VAR = 10)$
All	WT	θ_2	$Normal(E = 0, VAR = 10)$
All	WT	σ_{min}	$Gamma(shape = 2, rate = 1)$
All	WT	θ'_0	$\theta_0 + \Delta_{\theta_0}$

Model	Time.series	Parameter	Prior
All	WT	Δ_{theta_0}	$LogNormal(E = log(IQ90_{WT}/2) - .5, VAR = 1)$
All	WT	θ'_1	$Normal(E = 0, VAR = 10)$
All	WT	θ'_2	$Normal(E = 0, VAR = 10)$
All	WT	σ_{max}	$Gamma(shape = 2, rate = 1)$
All	WT	σ	$Gamma(shape = 2, rate = 1)$
M_1	WT	ρ	$Beta(\alpha = 5, \beta = 2)$
M_2	WT	δ	$Normal(E = 0, VAR = 1)$
M_2	WT	γ	$Normal(E = 0, VAR = 1)$

7.2 Time series used as application cases

Figure 8: Times series or raw data from the Rough and Scorff rivers used in this study.

7.3 Fitted α and β AT and Q time series

Figure 9: AT α and β parameters posterior distributions from the fits of the models.

Figure 10: LFL α and β parameters posterior distributions from the fits of the models.

Click here to access/download;Figure;Fig.1.map.tif

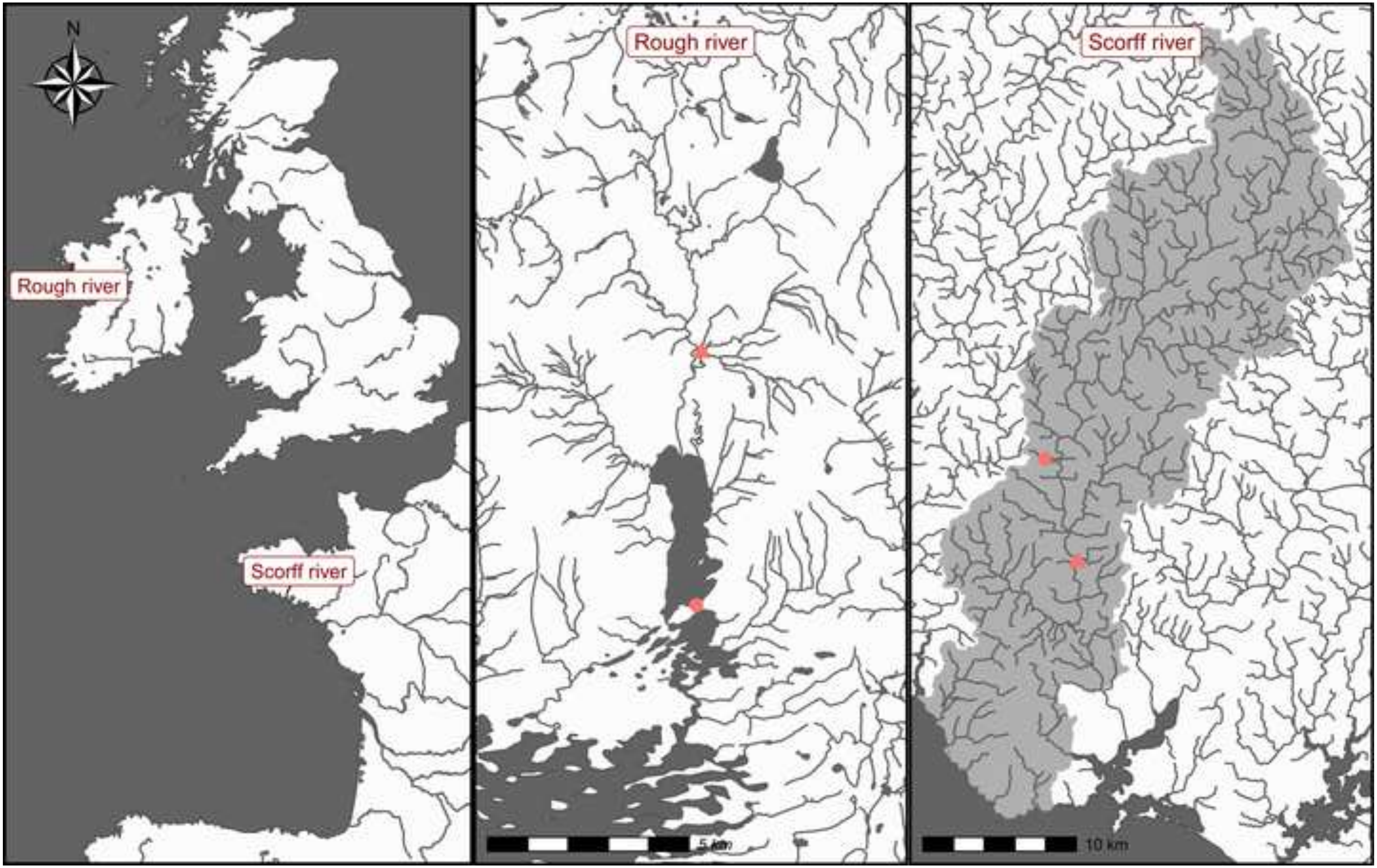

Click here to access/download;Figure;Fig.2.tif

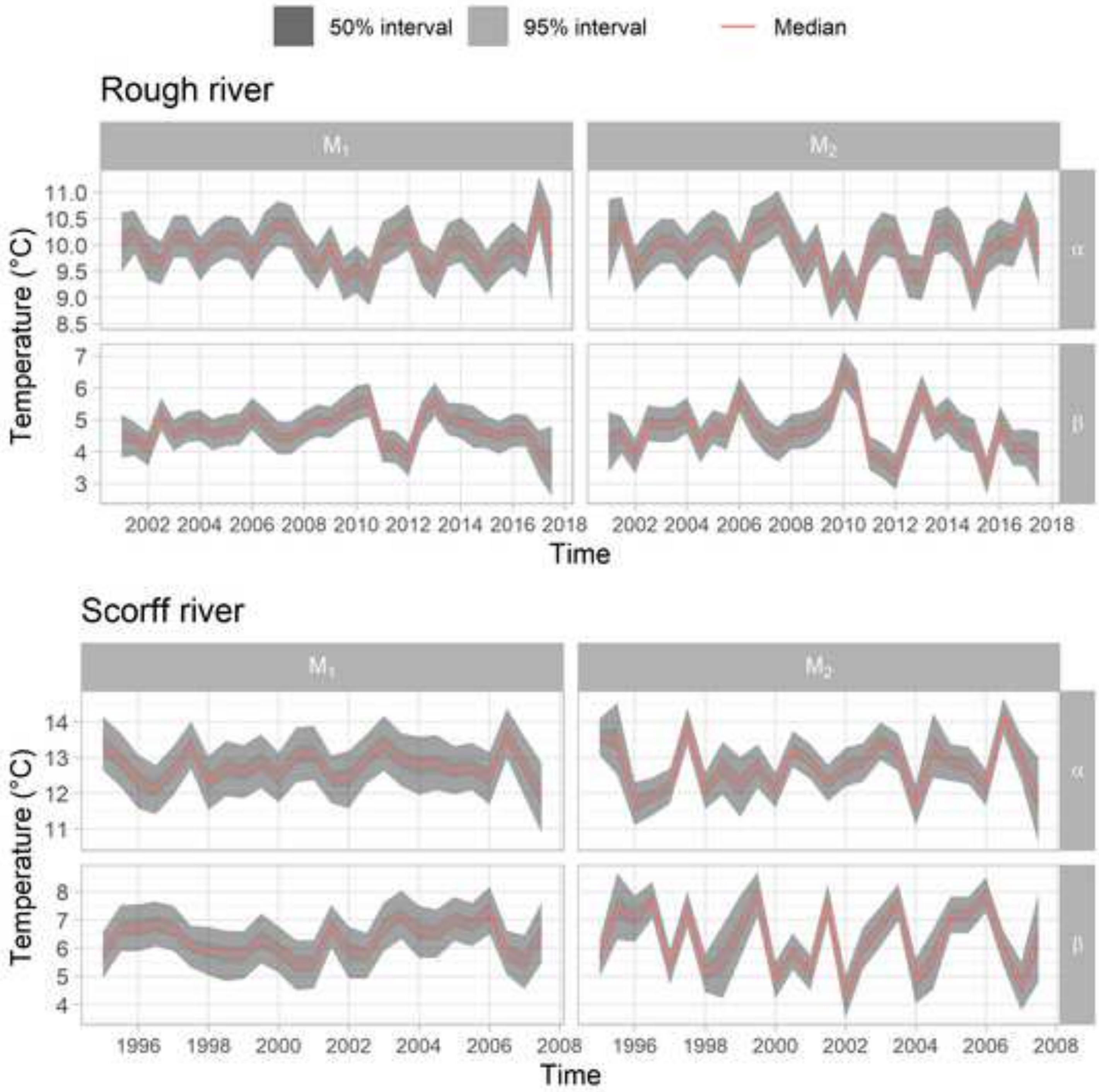

Click here to access/download;Figure;Fig.3.tif

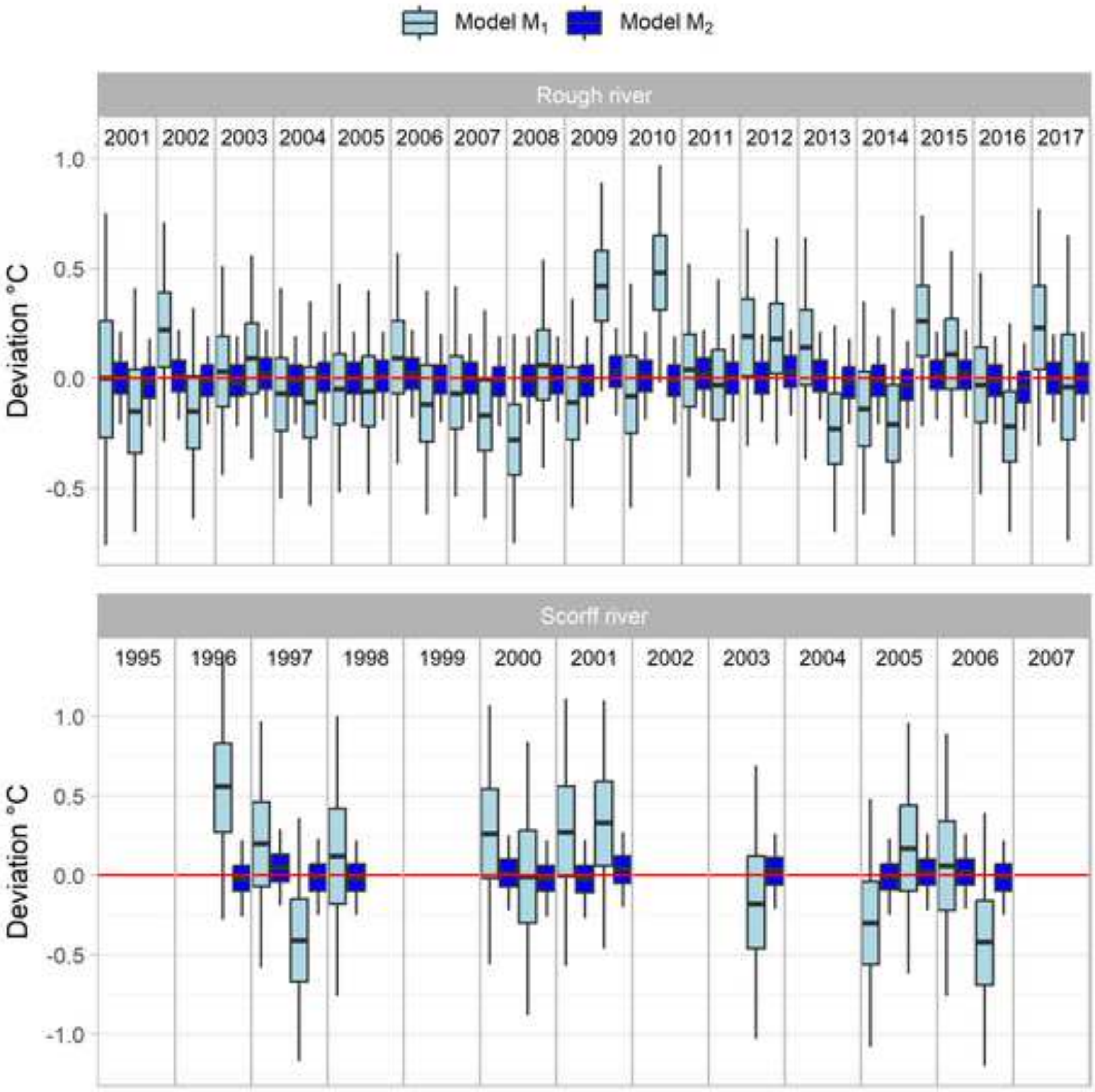

Click here to access/download;Figure;Fig.4.tif

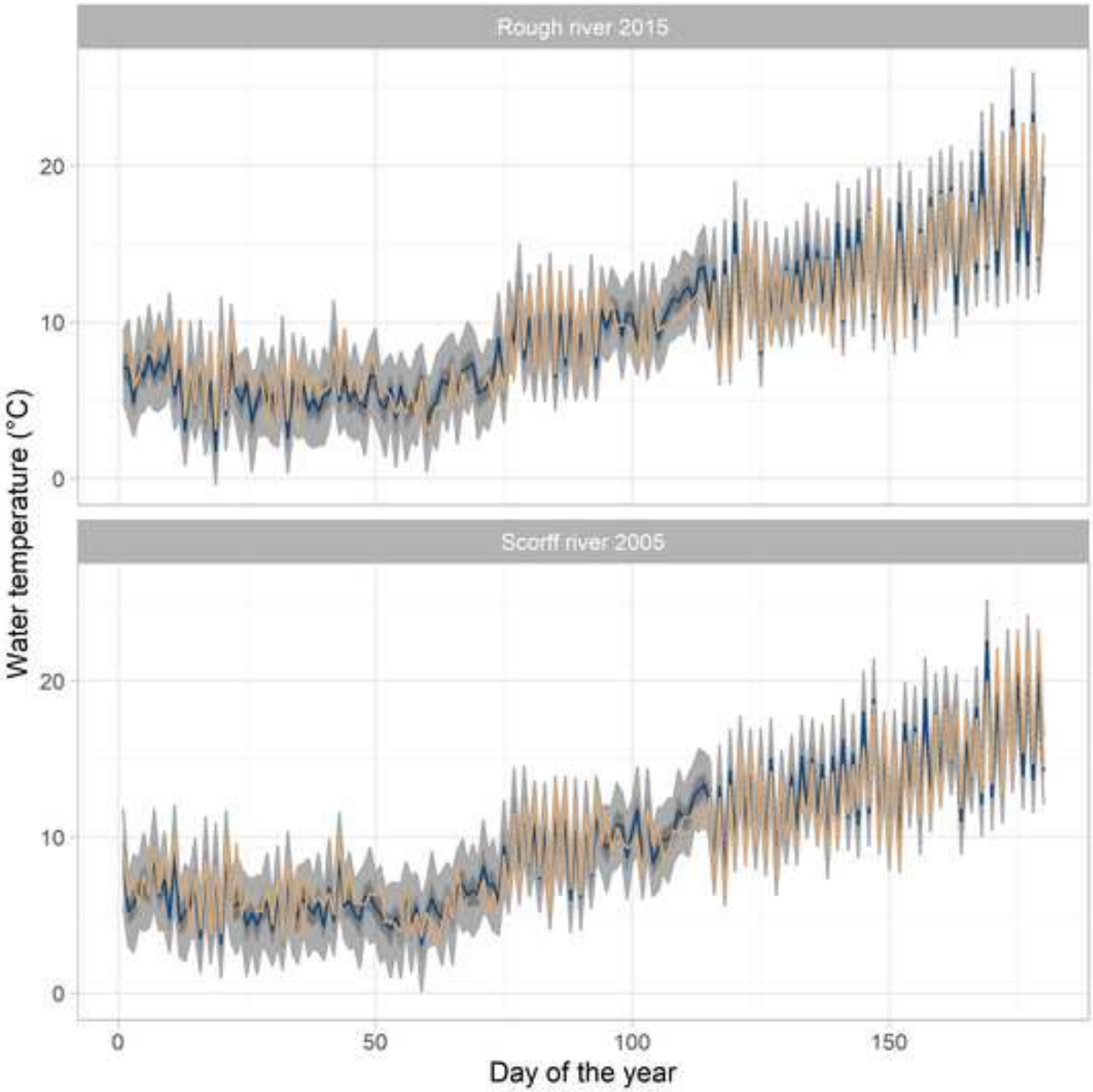

Click here to access/download;Figure;Fig.5.tif

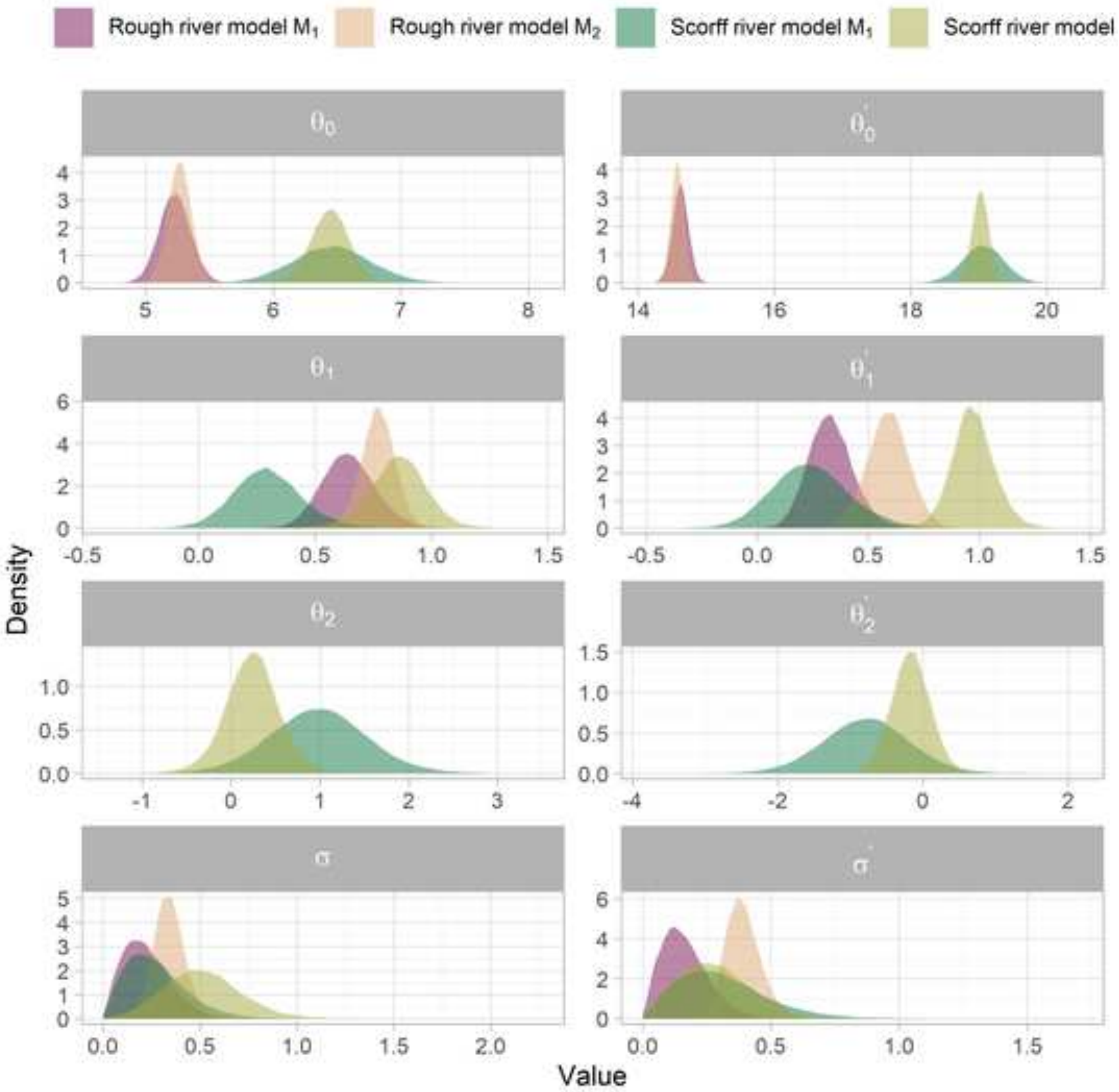

Click here to access/download;Figure;Fig.6.tif

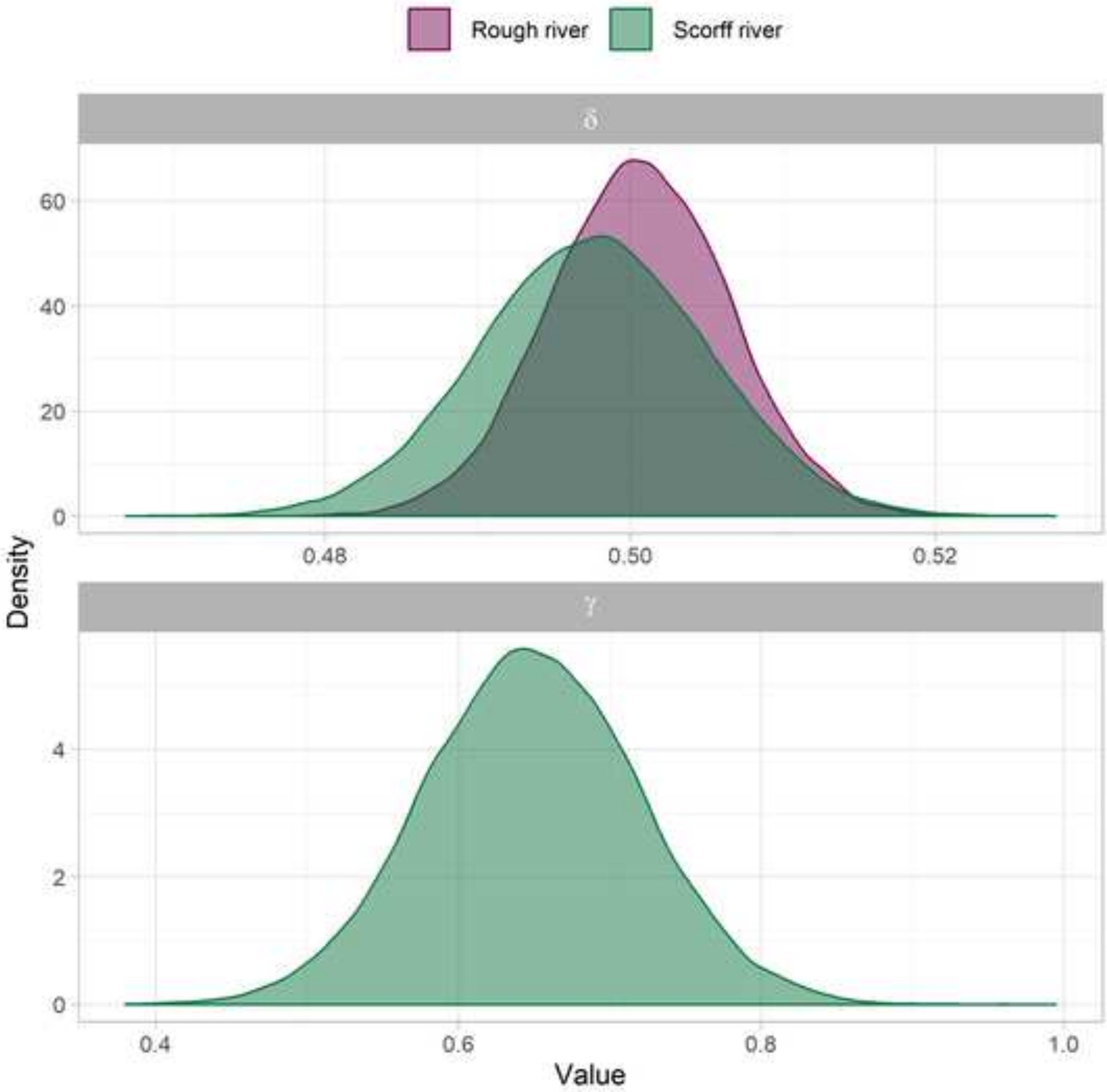

Click here to access/download;Figure;Fig.7.tif

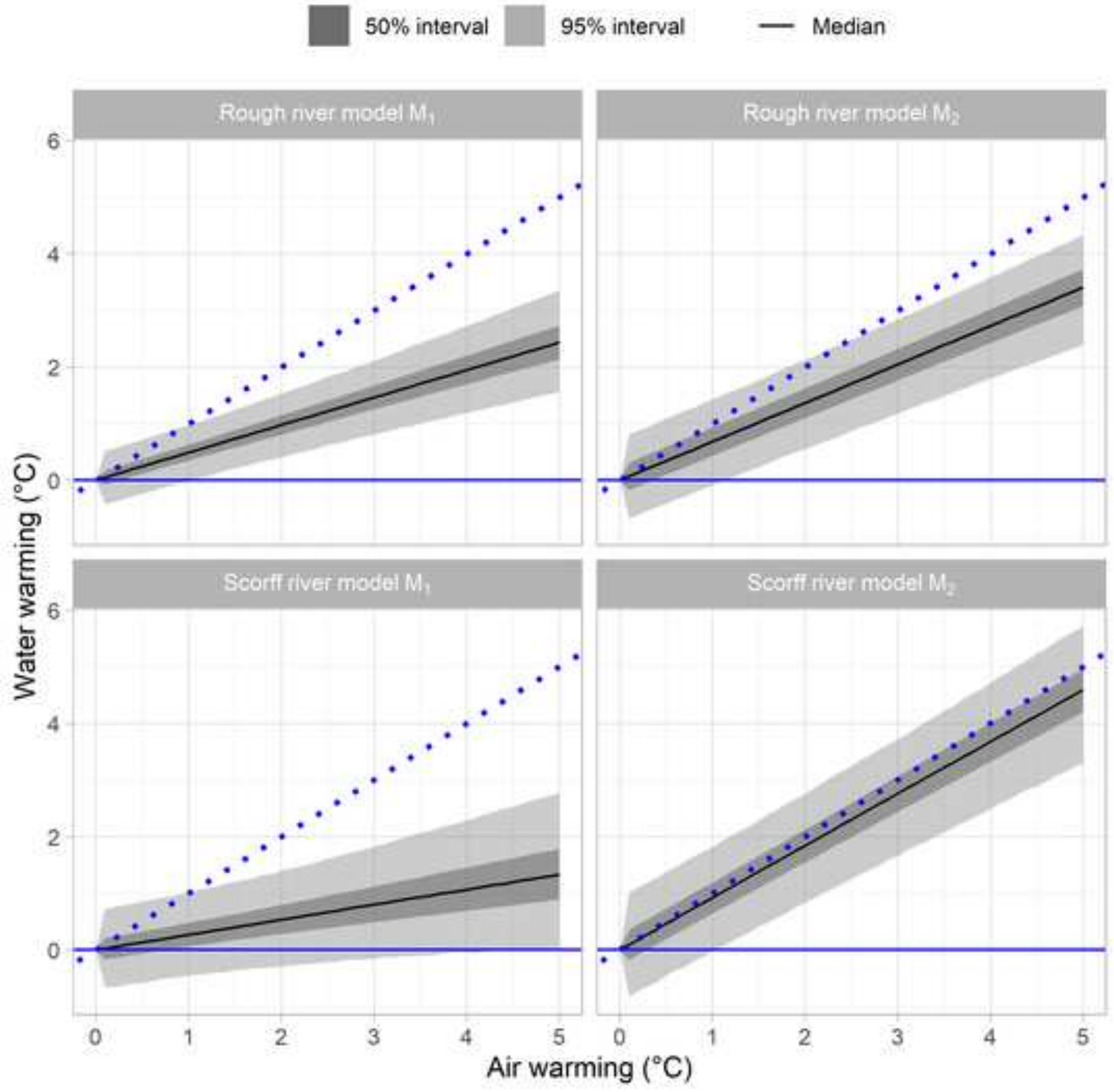

Click here to access/download;Figure;Fig.8.tif

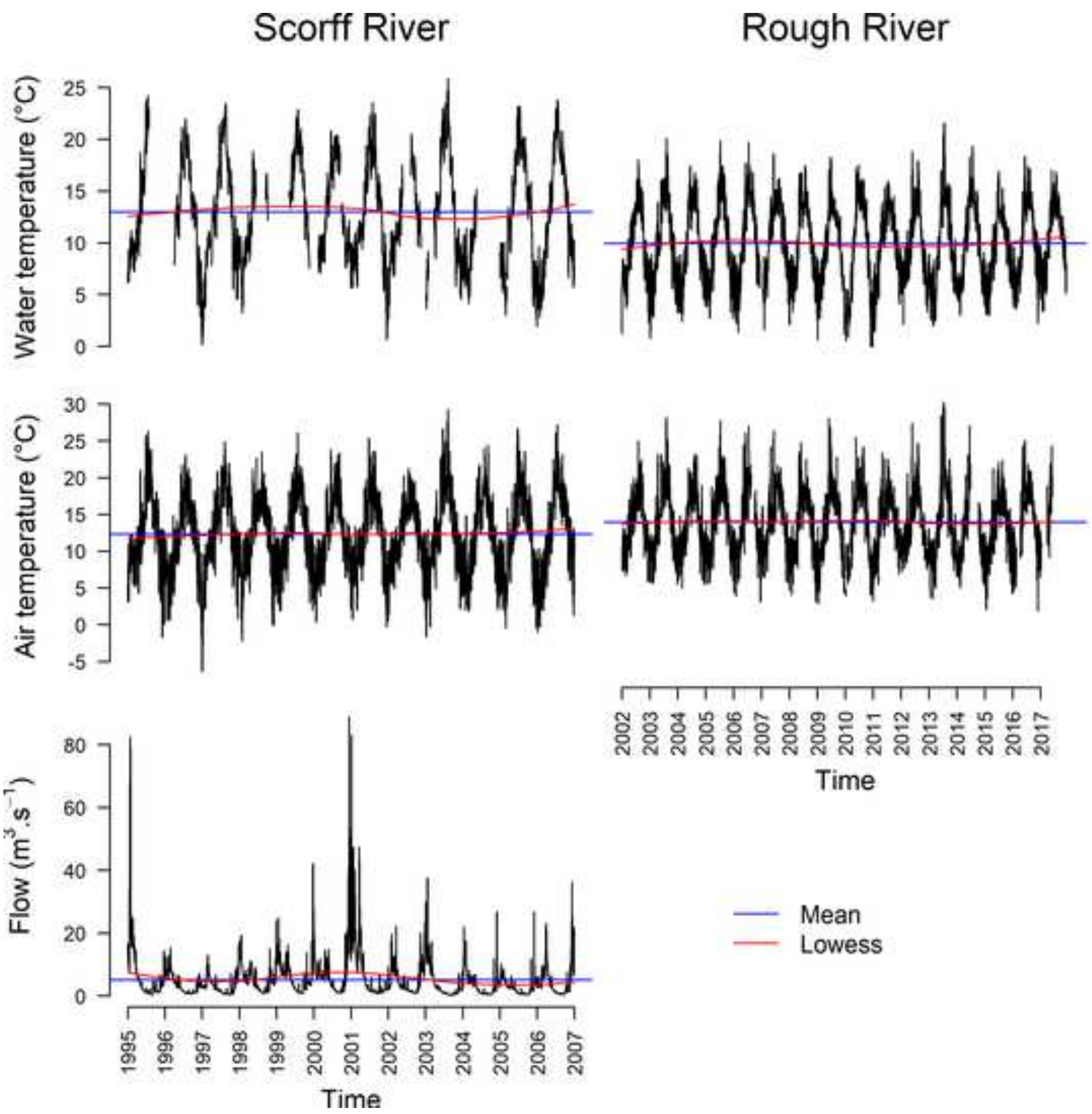

Click here to access/download;Figure;Fig.9.tif

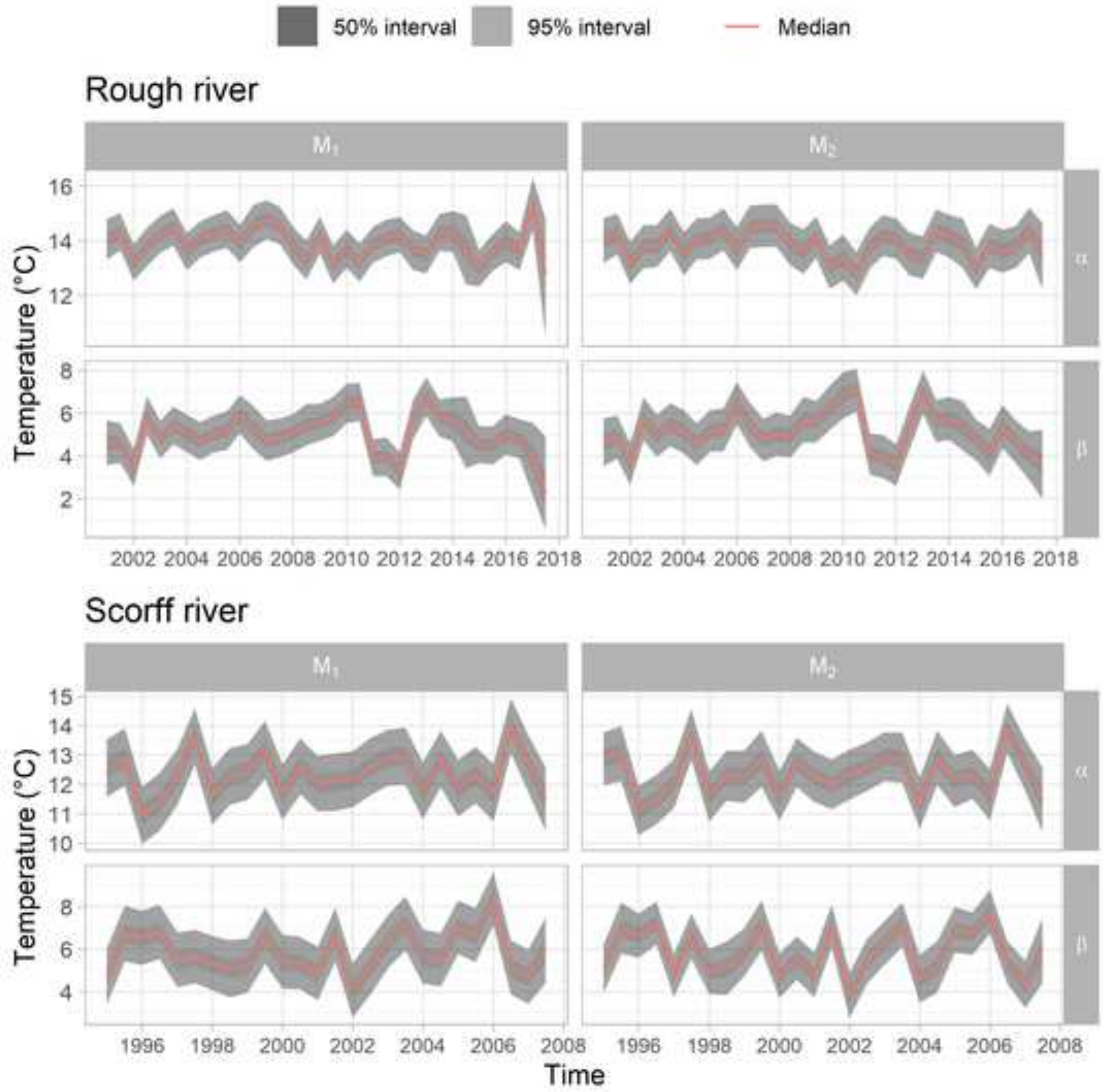

Click here to access/download;Figure;Fig.10.tif

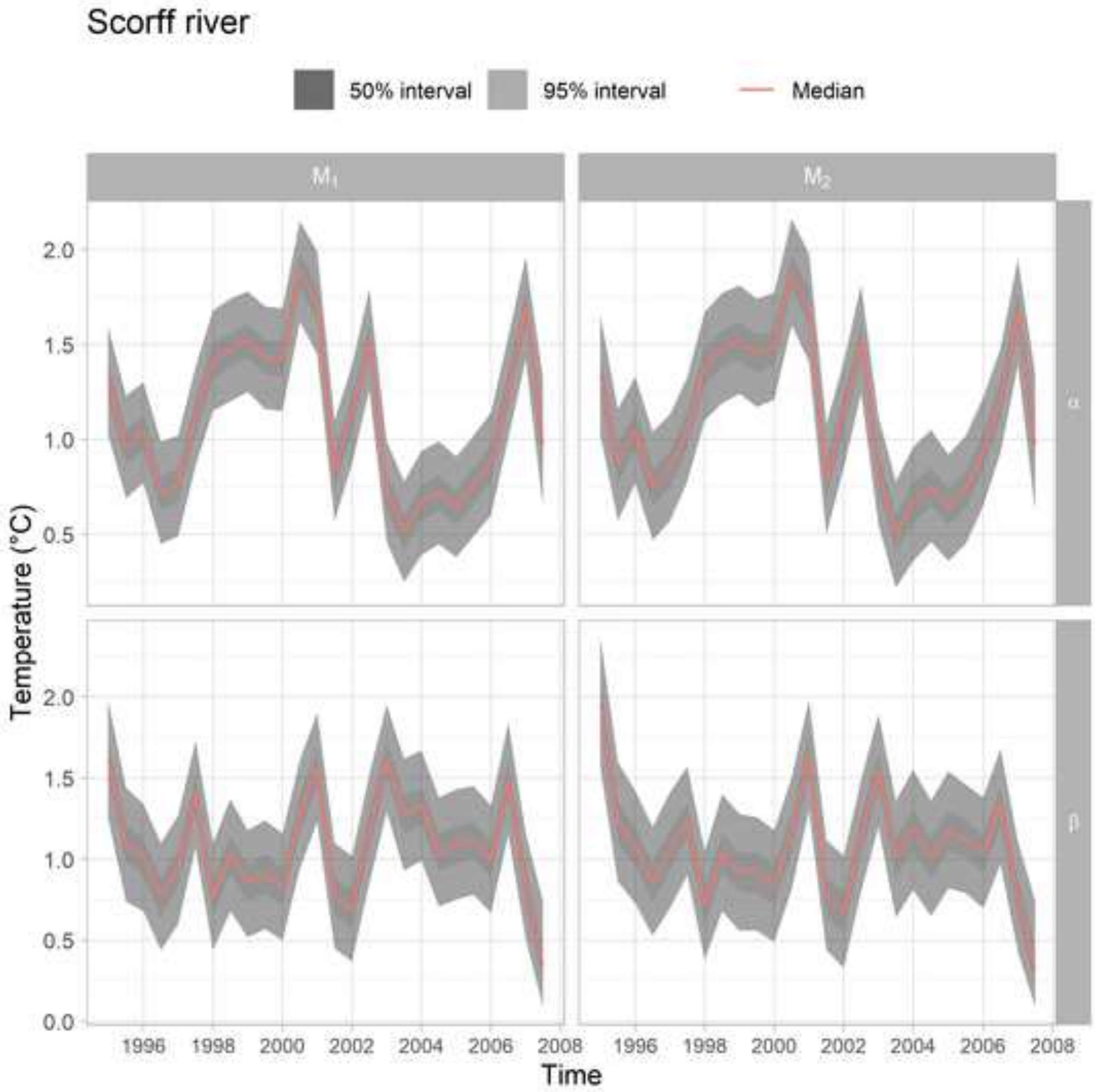

Supplement: Attachment — Submitted filename: PONE-D-23-03522.pdf [file pone.0291239.s002.pdf]
