# Supplementary material for: Simple Bayesian reconstruction and forecasting of stream water temperature for ecologists—A tool using air temperature, optionally flow, in a time series decomposition approach
Source: PLoS One. 2023 Sep 18;18(9):e0291239. doi: 10.1371/journal.pone.0291239 (PMC10506714; doi:10.1371/journal.pone.0291239)
Supplement: Attachment — Submitted filename: Plos One Comments Ecology.docx [file pone.0291239.s003.docx]

**Review comments**

The work describes the potential application of a conceptualized model for forecasting the global warming. The text describes the impact of global warming on ecological communities and ecosystems worldwide, with a focus on freshwater species. Several works have been reported in this line, and the present work can substantiate to existing knowledge. The overall content is interesting and publishable as it lies within the scope of the journal. At this point, I am against publication of the work, however, suggestions for its improvement are appended.

**Abstract**

Overall, this is a well-written and informative piece. You provide a clear and concise summary of the four practical steps for mitigating the impacts of global warming on wildlife and highlight the challenges associated with obtaining good temperature data for stream ecologists and managers. You then explain how your expanded hierarchical Bayesian model can help address this data gap by providing daily time series of water temperature and improving our understanding of the uncertainties in warming trends.

Writing style is clear and easy to follow, and you provide specific examples and details to illustrate your points. Additionally, you include technical terms and concepts, but you explain them in a way that is accessible to a general audience.

One suggestion for improvement would be to include more specific details about the case study rivers you used to test the model. For example, you could provide more information about the location of the rivers, their size and characteristics, and any relevant environmental or management factors that might influence their water temperatures. This would help readers better understand the context and relevance of your findings.

Due to the high susceptibility of these species to temperature changes and the limited availability of water temperature time series data, there is a need for models to complete and forecast water temperature. The limitations of existing models are discussed, and a Bayesian hierarchical approach is proposed as a solution. The extended model presented in the text, which includes daily temperature and modeling of daily deviations, is compared to the previous model on two rivers with different conditions. The text provides valuable information and is well-structured, with clear explanations of the different types of models and their advantages and disadvantages.

**Discussion**

This section discusses the improvements made to a model of water temperature (WT) forecasting using a time series decomposition-based approach. The new model, referred to as M2, takes into account daily variation in addition to seasonal variations, resulting in better WT forecasting abilities. The RMSE decreased by about 30% on the Rough River and 40% on the Scorff River, indicating a better fit. The use of an AR1 structure to model WT residuals in the previous model (M1) was found to be overfitting, resulting in poorer long-term hindcasts or projected forecasts.

The new model also suggests that daily variation in flow may be more important to WT than seasonal variation. The impact of daily flow variations on WT has practical consequences, such as daily management of flow to avoid extremely low and high flow, which might be useful in the context of climate change adaptation. The model also highlights that WT warming should track that of air temperature (AT) more closely than predicted in the previous model.

However, the overall uncertainty associated with WT warming is high, and this must be accounted for in decision making. The approach developed in this study makes it possible to integrate this uncertainty into a probabilistic framework. Further extensions of the model are possible, and it is suggested that the model could be applied to other rivers to determine if the behavior observed is consistent.

My overall view for this submission is revise with minor modifications.
