# Supplementary material for: Simple Bayesian reconstruction and forecasting of stream water temperature for ecologists—A tool using air temperature, optionally flow, in a time series decomposition approach
Source: PLoS One. 2023 Sep 18;18(9):e0291239. doi: 10.1371/journal.pone.0291239 (PMC10506714; doi:10.1371/journal.pone.0291239)
Supplement: Attachment — Submitted filename: reply.table.docx [file pone.0291239.s004.docx]

# Editors comments reply table

1. Please ensure that your manuscript meets PLOS ONE's style requirements, including those for file naming. The PLOS ONE style templates can be found at

<https://journals.plos.org/plosone/s/file?id=wjVg/PLOSOne_formatting_sample_main_body.pdf> and

<https://journals.plos.org/plosone/s/file?id=ba62/PLOSOne_formatting_sample_title_authors_affiliations.pdf>

Done accordingly to the templates provided

2. We note that you have stated that you will provide repository information for your data at acceptance. Should your manuscript be accepted for publication, we will hold it until you provide the relevant accession numbers or DOIs necessary to access your data. If you wish to make changes to your Data Availability statement, please describe these changes in your cover letter and we will update your Data Availability statement to reflect the information you provide.

We updated the cover letter accordingly. In particular, the datasets used now benefit from a DOI or we provide links to request/download the data on the related French environmental services databases. Those links are also provided onto the GITHub repository hosting our model code.

3. Please ensure that you refer to Figures 8, 9 and 10 in your text as, if accepted, production will need this reference to link the reader to the figure.

There now referred to as Figs S1-S3 and mentioned in the results section.

4. We note you have included a table to which you do not refer in the text of your manuscript. Please ensure that you refer to Table 3 in your text; if accepted, production will need this reference to link the reader to the Table.

Now called table S1 and referred to within the Material and method section (see end of Bayesian computation subsection)

5. We note that Figure 1 in your submission contain map images which may be copyrighted. All PLOS content is published under the Creative Commons Attribution License (CC BY 4.0), which means that the manuscript, images, and Supporting Information files will be freely available online, and any third party is permitted to access, download, copy, distribute, and use these materials in any way, even commercially, with proper attribution. For these reasons, we cannot publish previously copyrighted maps or satellite images created using proprietary data, such as Google software (Google Maps, Street View, and Earth). For more information, see our copyright guidelines: <http://journals.plos.org/plosone/s/licenses-and-copyright>.

We require you to either (1) present written permission from the copyright holder to publish these figures specifically under the CC BY 4.0 license, or (2) remove the figures from your submission:

   a. You may seek permission from the original copyright holder of Figure 1 to publish the content specifically under the CC BY 4.0 license.

 We recommend that you contact the original copyright holder with the Content Permission Form (<http://journals.plos.org/plosone/s/file?id=7c09/content-permission-form.pdf>) and the following text:

“I request permission for the open-access journal PLOS ONE to publish XXX under the Creative Commons Attribution License (CCAL) CC BY 4.0 (<http://creativecommons.org/licenses/by/4.0/>). Please be aware that this license allows unrestricted use and distribution, even commercially, by third parties. Please reply and provide explicit written permission to publish XXX under a CC BY license and complete the attached form.”

Please upload the completed Content Permission Form or other proof of granted permissions as an "Other" file with your submission.

 In the figure caption of the copyrighted figure, please include the following text: “Reprinted from [ref] under a CC BY license, with permission from [name of publisher], original copyright [original copyright year].”

  b. If you are unable to obtain permission from the original copyright holder to publish these figures under the CC BY 4.0 license or if the copyright holder’s requirements are incompatible with the CC BY 4.0 license, please either i) remove the figure or ii) supply a replacement figure that complies with the CC BY 4.0 license. Please check copyright information on all replacement figures and update the figure caption with source information. If applicable, please specify in the figure caption text when a figure is similar but not identical to the original image and is therefore for illustrative purposes only.

The figure mentioned shows maps of two watersheds as well as a general map showing the rivers location within Europe. All the shapefiles used to produce figure are coming from sources compatible with the CC BY 4.0 license used by PLOSONE :

1. GADM data (<https://gadm.org/license.html>).
   a. **“The data are freely available for academic use and other non-commercial use. Redistribution or commercial use is not allowed without prior permission.**
      Using the data to create maps for publishing of academic research articles is allowed. Thus you can use the maps you made with GADM data for figures in articles published by PLoS, Springer Nature, Elsevier, MDPI, etc. You are allowed (but not required) to publish these articles (and the maps they contain) under an open license such as CC-BY as is the case with PLoS journals and may be the case with other open access articles. **Data for the following countries is covered by a a different license** **Austria**: Creative Commons Attribution-ShareAlike 2.0 (source: Government of Ausria)”
2. Environmental Protection Agency Ireland, water flow network (<https://gis.epa.ie/geonetwork/srv/api/records/c4043e19-38ec-4120-a588-8cd01ac94a9c>)
   a. “Data is for public use under Creative Commons CC-By 4.0”
3. BD carthage, French river network (<https://geo.data.gouv.fr/fr/datasets/05ca9c8713d19f758d4acd9c37ce4a571b4f5ae5>)
   a. “Licence ouverte / Open Licence”, compatible with CC-by type licences (<https://www.etalab.gouv.fr/wp-content/uploads/2017/04/ETALAB-Licence-Ouverte-v2.0.pdf>)

Sources are now acknowledged within the Figure legend, as is the R package used for the drawing.

Additional Editor Comments:

The article needs substantial revision in language in terms of grammatical and phrases related mistakes.

We took note of the editor’scomment. Dr. de Eyto, who is a native speaker, polished the writing for this new version whilst also trying to preserve the voice of the first author who is a native french speaker. Dr. de Eyto feels it is important to not erase the natural voice of the first author, so long as it does not impede the flow of the manuscript, and is technically correct (which is agreed on by the reviewer). We believe that is should now be acceptable for publication.

# Reviewer 1 comments reply table

We put the entire review here, but the only comment to address is in paragraph 4. Please see our answer there.

The work describes the potential application of a conceptualized model for forecasting the global warming. The text describes the impact of global warming on ecological communities and ecosystems worldwide, with a focus on freshwater species. Several works have been reported in this line, and the present work can substantiate to existing knowledge. The overall content is interesting and publishable as it lies within the scope of the journal. At this point, I am against publication of the work, however, suggestions for its improvement are appended.

**Abstract**

Overall, this is a well-written and informative piece. You provide a clear and concise summary of the four practical steps for mitigating the impacts of global warming on wildlife and highlight the challenges associated with obtaining good temperature data for stream ecologists and managers. You then explain how your expanded hierarchical Bayesian model can help address this data gap by providing daily time series of water temperature and improving our understanding of the uncertainties in warming trends.

Writing style is clear and easy to follow, and you provide specific examples and details to illustrate your points. Additionally, you include technical terms and concepts, but you explain them in a way that is accessible to a general audience.

One suggestion for improvement would be to include more specific details about the case study rivers you used to test the model. For example, you could provide more information about the location of the rivers, their size and characteristics, and any relevant environmental or management factors that might influence their water temperatures. This would help readers better understand the context and relevance of your findings.

We agree the text about the watershed description is concise but it comes with a map as wells as a complementary table (Table 1) to allow for quick and easy side by side comparison of the rivers’ characteristics. In addition to the elements suggested by the reviewer, the table also includes statistics about the watershed land use and elevation. To the best of our knowledge, these rivers are not impacted by hydromorphological changes. Overall, we provided a fair amount of information which is on par with what is usually done within the literature cited.

Due to the high susceptibility of these species to temperature changes and the limited availability of water temperature time series data, there is a need for models to complete and forecast water temperature. The limitations of existing models are discussed, and a Bayesian hierarchical approach is proposed as a solution. The extended model presented in the text, which includes daily temperature and modeling of daily deviations, is compared to the previous model on two rivers with different conditions. The text provides valuable information and is well-structured, with clear explanations of the different types of models and their advantages and disadvantages.

**Discussion**

This section discusses the improvements made to a model of water temperature (WT) forecasting using a time series decomposition-based approach. The new model, referred to as M2, takes into account daily variation in addition to seasonal variations, resulting in better WT forecasting abilities. The RMSE decreased by about 30% on the Rough River and 40% on the Scorff River, indicating a better fit. The use of an AR1 structure to model WT residuals in the previous model (M1) was found to be overfitting, resulting in poorer long-term hindcasts or projected forecasts.

The new model also suggests that daily variation in flow may be more important to WT than seasonal variation. The impact of daily flow variations on WT has practical consequences, such as daily management of flow to avoid extremely low and high flow, which might be useful in the context of climate change adaptation. The model also highlights that WT warming should track that of air temperature (AT) more closely than predicted in the previous model.

However, the overall uncertainty associated with WT warming is high, and this must be accounted for in decision making. The approach developed in this study makes it possible to integrate this uncertainty into a probabilistic framework. Further extensions of the model are possible, and it is suggested that the model could be applied to other rivers to determine if the behavior observed is consistent.

My overall view for this submission is revise with minor modifications.
